# Elevated Histone Lactylation Mediates Ferroptosis Resistance in Endometriosis Through the METTL3‐Regulated HIF1A/HMOX1 Signaling Pathway

**DOI:** 10.1002/advs.202408220

**Published:** 2025-06-10

**Authors:** Zongwen Liang, Jinming Liu, Yanling Gou, Honglin Wang, Zhi Li, Yingying Cao, Huiyan Zhang, Ruru Bai, Zongfeng Zhang

**Affiliations:** ^1^ Department of Obstetrics and Gynecology The Second Affiliated Hospital of Harbin Medical University Harbin 150086 China

**Keywords:** endometriosis, ferroptosis, hif1a, histone lactylation, hmox1, mettl3

## Abstract

Endometriosis (EMs) is a chronic gynecologic condition characterized by the growth of endometrial stromal and glandular tissue outside the uterine cavity of unknown etiology. Currently, ferroptosis resistance, increased glycolysis, and increased lactate production are identified in EMs. Histone lactylation is a lactate‐derived posttranslational modification that is recognized primarily for its role in epigenetic regulation. In this study, it is demonstrated that increased histone lactylation contributes to ferroptosis resistance in ectopic endometrial stromal cells (EESCs). Mechanistically, histone lactylation mediates ferroptosis resistance through the hypoxia‐inducible factor 1 alpha (HIF1A)/heme oxygenase 1 (HMOX1) signaling pathway, which is regulated by methyltransferase like 3 (METTL3). In vivo experiments reveal that combination therapy with 2‐deoxy‐D‐glucose (2‐DG) and erastin is more effective for the treatment of EMs. Together, the findings provide a theoretical basis for the pathogenesis of EMs and suggest that a combined treatment that inhibits histone lactylation and induces ferroptosis is an effective treatment for EMs.

## Graphical abstract

This study reveals that histone lactylation, a modification derived from lactate, plays a crucial role in ferroptosis resistance in ectopic endometrial stromal cells. This resistance is mediated via the HIF1A/HMOX1 pathway regulated by METTL3. In vivo, a combination of 2‐DG and erastin shows promise as an effective treatment for endometriosis.

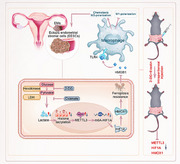

## 1 Introduction

Endometriosis (EMs) is a chronic gynecological disease characterized by the presence of endometrial stroma and glands outside the uterine cavity, leading to pelvic pain and infertility. It affects 6%–10% of women of reproductive age, with an unclear pathogenesis.^[^ ^1^ ^]^ The primary treatments for this condition are surgical intervention and pharmacotherapy. However, the invasiveness of surgery and the side effects of medical treatment greatly limit its clinical application.^[^ ^2^, ^3^, ^4^ ^]^ Therefore, understanding the pathogenesis of EMs and identifying effective treatments are crucial for prompt and accurate therapy.

Ferroptosis is an iron‐dependent form of programmed cell death characterized by glutathione (GSH) depletion and lethal lipid peroxide accumulation that differs from apoptosis and necrosis.^[^ ^5^ ^]^ Iron overload, resulting from periodic hemorrhage in ectopic lesions, is a key characteristic of endometriosis.^[^ ^6^ ^]^ Ferroptosis induced by iron overload has been demonstrated in ectopic endometrial stromal cells (EESCs) exposed to endometrioma cyst fluid.^[^ ^7^ ^]^ However, endometriotic cells often exhibit increased resistance to ferroptosis, allowing their survival, progression, and establishment of endometriotic lesions. Resistance to ferroptosis may serve as a hallmark of endometriosis.^[^ ^8^, ^9^ ^]^ The underlying mechanisms of ferroptosis resistance in endometriosis remain inadequately explored and understood.

The Warburg effect refers to the phenomenon in which tumor cells prefer glycolysis over oxidative phosphorylation as their primary source of energy to meet their rapid growth and proliferation needs under both hypoxic and aerobic conditions.^[^ ^10^ ^]^ This phenomenon is prevalent in tumors and has also been described in EMs lesions. Many vital glycolytic enzymes, such as 6‐phosphofructo‐2‐kinase/fructose‐2,6‐biphosphatase 3 ^[^ ^11^ ^]^ and pyruvate kinase M2,^[^ ^12^ ^]^ are highly expressed and contribute to the progression of EMs. Studies have demonstrated that aurora kinase A enhances glycolysis and the development of ovarian endometriosis through ERβ.^[^ ^13^ ^]^ In EMs, glycolysis generates substantial amounts of lactate, creating an acidic microenvironment that promotes cell invasion, cell metastasis, and immunosuppression.^[^ ^14^ ^]^ Zhang et al. demonstrated that lactate accumulation promotes lactylation of lysine (K) residues of histones, directly enhancing gene transcription in chromatin.^[^ ^15^ ^]^ Although lactate has been reported to induce tumor resistance to ferroptosis,^[^ ^16^ ^]^ whether histone lactylation is involved in the regulation of ferroptosis resistance in EMs has not been investigated. N6‐Methyladenine (m6A) is the most prevalent post‐transcriptional RNA modification in mammals and is characterized by dynamic and reversible regulation.^[^ ^17^ ^]^ Recent studies have indicated that histone lactylation mediates the modification of m6A‐associated genes and subsequently regulates tumor development.^[^ ^18^, ^19^ ^]^ Several studies have revealed that aberrant m6A levels regulate ferroptosis under different pathological conditions.^[^ ^20^, ^21^, ^22^ ^]^

In the present study, we revealed a potential mechanism of ferroptosis resistance in EMs mediated by methyltransferase like 3 (METTL3). We demonstrated that increased histone lactylation regulates ferroptosis resistance in EESCs through the hypoxia‐inducible factor 1 alpha (HIF1A)/heme oxygenase 1 (HMOX1) signaling pathway modulated by METTL3. Finally, in a mouse model of EMs, treatment with 2‐deoxy‐D‐glucose (2‐DG) combined with erastin significantly reduced the volume of ectopic lesions more than treatment with 2‐DG or erastin alone did. Together, our findings indicate that increased histone lactylation promotes ferroptosis resistance and that targeting histone lactylation and ferroptosis might be an effective strategy for treating EMs.

## 2 Results

### 2.1 Histone Lactylation Levels are Increased in EESCs

**Figure 1:**
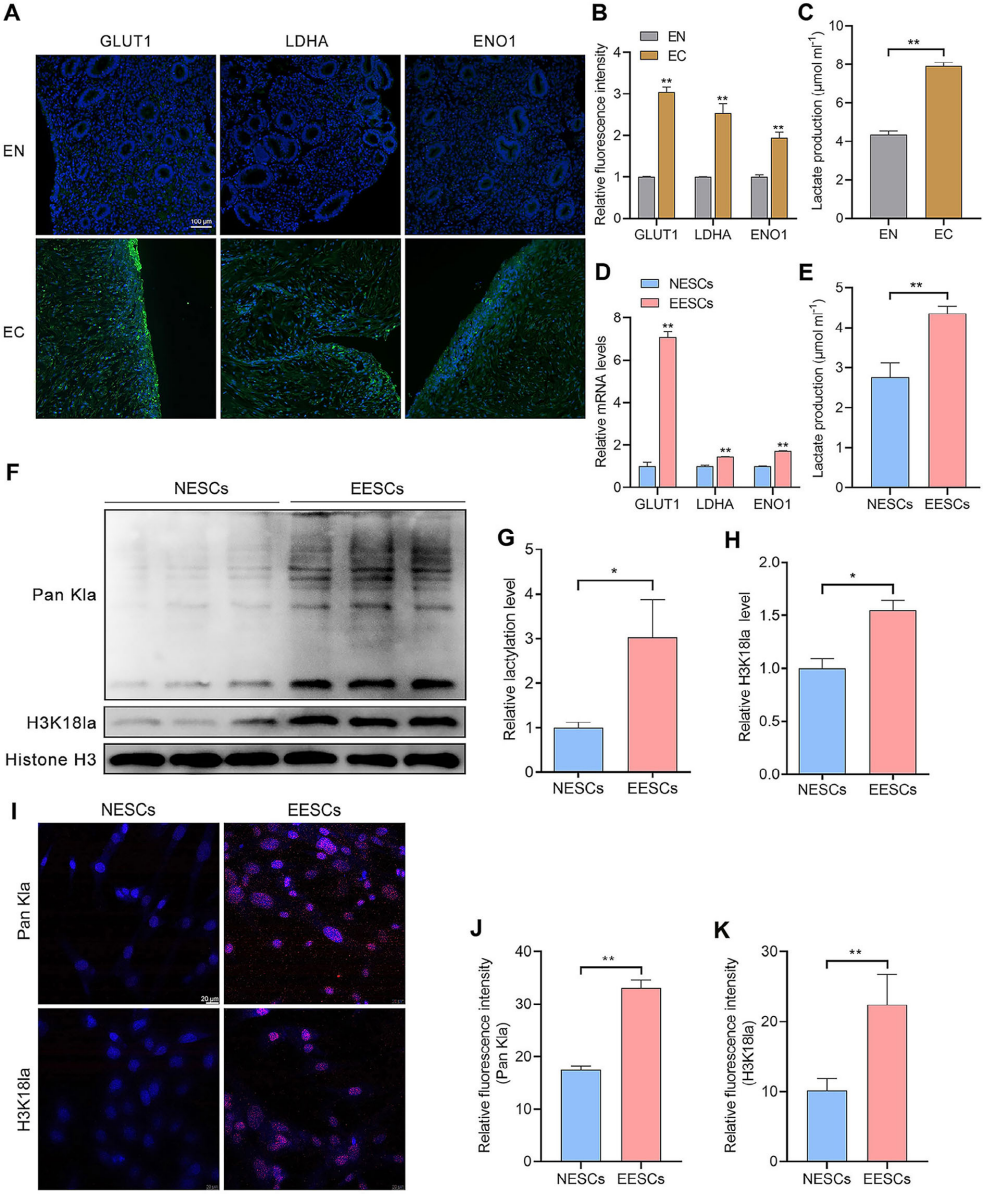
Histone lactylation levels are increased in EESCs. A) Representative IF images of glycolysis‐related marker proteins (green) in EN (*n* = 10) and EC (*n* = 10) tissues, Nuclei were stained with DAPI (blue). Scale bar, 100 µm. B) Quantitative fluorescence intensity of glucose transporter 1 (GLUT1), lactate dehydrogenase A (LDHA), alpha‐enolase (ENO1). C) Lactate levels in EN (*n* = 10) and EC (*n* = 10) tissues were detected using lactate assay kits. D) The expression of glycolysis‐related genes in NESCs and EESCs was assessed by qRT‐PCR. E) Lactate levels in NESCs and EESCs were detected using lactate assay kits. F–H) Global lactylation and H3K18la levels in NESCs and EESCs were determined by western blot assays. I) Representative IF images of pan‐Kla and H3K18la (red) in NESCs and EESCs, Nuclei were stained with DAPI (blue). Scale bar, 20 µm. J) Quantitative fluorescence intensity of pan Kla. K) Quantitative fluorescence intensity of H3K18la. Error bars represent the mean ± SD. Statistical analyses were performed using a two‐tailed Student's *t‐*test. NS no significance; ^*^ *p* < 0.05 and ^**^ *p* < 0.01.

As lactylation is a recently identified post‐translational modification (PTM) induced by lactate, its role in EMs remains largely unexplored. In the present study, immunofluorescence (IF) analysis revealed significant upregulation of glycolysis‐related marker proteins in ectopic endometrial (EC) tissues compared to normal endometrial (EN) tissues (**Figure** **1**A,B). Correspondingly, lactate quantification assays showed a substantial rise in lactate levels in EC tissues relative to EN tissues (Figure 1C). At the cellular level, quantitative reverse transcription polymerase chain reaction (qRT‐PCR) analysis confirmed a similar pattern, with significantly increased expression of glycolysis‐related genes in EESCs compared to NESCs (Figure 1D). Furthermore, lactate levels were markedly elevated in EESCs relative to those in NESCs (Figure 1E). Importantly, western blot analysis indicated a pronounced increase in global lactylation levels in EESCs compared to NESCs (Figure 1F,G). Notably, the predominant band in the western blot assays was near 17 kDa, possibly representing histone H3. Therefore, we investigated whether the above phenomenon was caused by hanalysis of varianceistone H3 lysine 18 lactylation (H3K18la). Similarly, the levels of H3K18la showed the same trend as the global lactylation levels did (Figure 1F–H). Subsequent IF staining revealed significantly higher levels of global lactylation in EESCs than in NESCs, and the lactylated proteins were mainly localized in the cell nucleus (Figure 1I,J). H3K18la showed a trend similar to that of the global lactylation levels (Figure 1I,K). These data indicate that the levels of histone lactylation were elevated in EMs, which is likely involved in the development of EMs.

### 2.2 Elevated Histone Lactylation Promotes Resistance to Ferroptosis in EESCs

**Figure 2:**
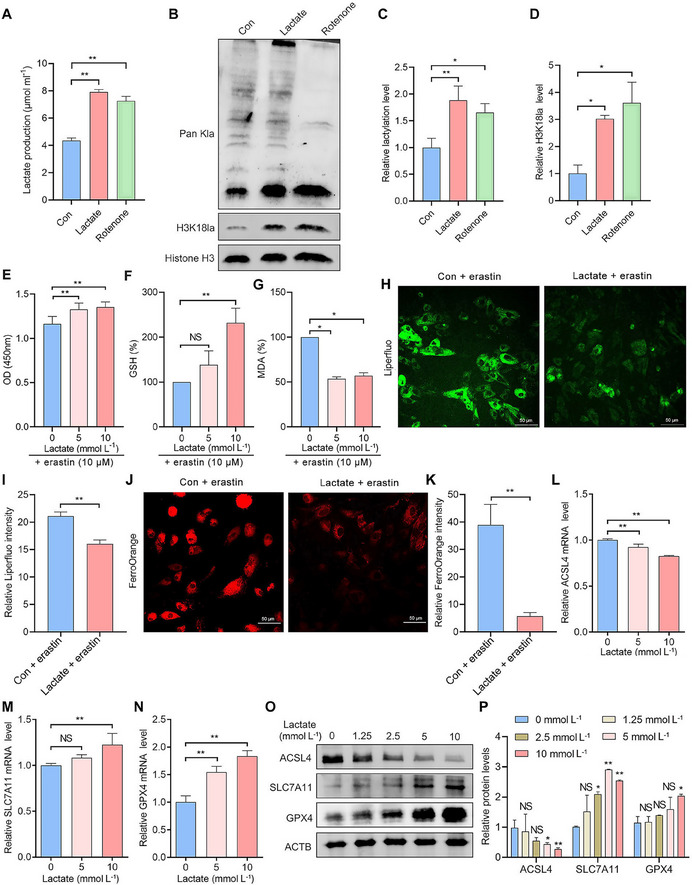
Elevated histone lactylation promotes ferroptosis resistance in EESCs. A) Intracellular lactate levels were measured using lactate assay kits after treatment with lactate or rotenone. B–D) Global lactylation and H3K18la levels in EESCs were assessed by western blot assays after treatment with lactate or rotenone. E–G) EESCs were stimulated with different concentrations of lactate (0, 5, and 10 mmol L^−1^) for 24 h after treatment with erastin (10 µm) for 24 h. Cell viability was determined by a CCK‐8 assay E). GSH levels were detected with a GSH assay kit F). Malondialdehyde (MDA) levels were detected with an MDA assay kit G). H–K) EESCs were stimulated with lactate (10 mmol L^−1^) for 24 h after erastin (10 µm) for 24 h. Intracellular lipid peroxidation levels were determined by Liperfluo staining (scale bar = 50 µm) H,I). Intracellular Fe^2+^ levels were assessed by FerroOrange staining (scale bar = 50 µm) J,K). L–N) qRT‐PCR was performed to detect ACSL4, SLC7A11, and GPX4 mRNA levels in EESCs after treatment with different concentrations of lactate (0, 5, and 10 mmol L^−1^) for 24 h. O,P) Western blot analysis was performed to detect ACSL4, SLC7A11, and GPX4 protein levels in EESCs after treatment with different concentrations of lactate (0, 1.25, 2.5, 5, and 10 mmol L^−1^) for 48 h. Error bars represent the mean ± SD. Two‐tailed Student's *t*‐test was used to compare the means of two groups; One‐way analysis of variance (ANOVA) was used to compare the means of more than two groups; NS no significant; ^*^ *p* < 0.05; ^**^ *p* < 0.01.

**Figure 3:**
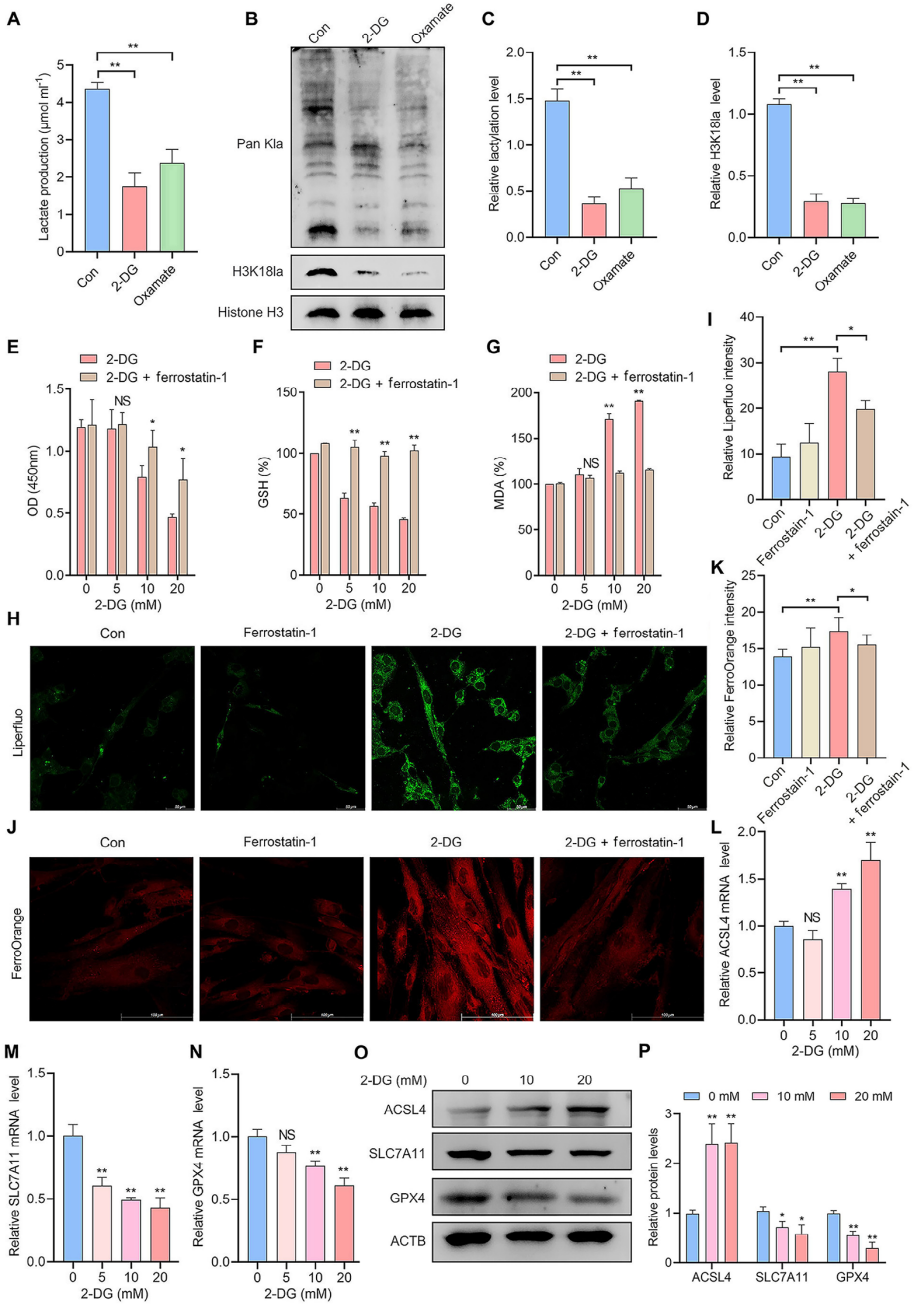
Inhibition of histone lactylation promotes ferroptosis in EESCs. A) Intracellular lactate levels were measured using lactate assay kits after treatment with 2‐DG and oxamate. B–D) Levels of global lactylation and H3K18la in EESCs were detected by western blotting after treatment with 2‐DG and oxamate. E–G) EESCs were stimulated with different concentrations of 2‐DG (0, 5, 10, and 20 mm) for 24 h, with or without the addition of ferrostatin‐1. Cell viability was assessed by CCK‐8 assay E). GSH levels were detected using a GSH assay kit F). MDA levels were detected using an MDA assay kit G). H,I) Intracellular lipid peroxidation levels in EESCs in the indicated groups were assessed by Liperfluo staining (scale bar = 50 µm). J,K) Intracellular Fe^2+^ levels in EESCs in the indicated groups were assessed by FerroOrange staining (scale bar = 100 µm). L–N) qRT‐PCR was performed to detect ACSL4, SLC7A11, and GPX4 mRNA levels in EESCs after treatment with different concentrations of 2‐DG (0, 5, 10, and 20 mm) for 24 h. O,P) Western blot analysis was performed to detect ACSL4, SLC7A11, and GPX4 protein levels in EESCs after treatment with different concentrations of 2‐DG (0, 10, and 20 mm) for 48 h. Error bars represent the mean ± SD. Two‐tailed Student's *t*‐test was used to compare the means of two groups; One‐way ANOVA was used to compare the means of more than two groups; NS indicates not statistically significant; ^*^ *p* < 0.05; ^**^ *p* < 0.01.

Building on our previous findings that ferroptosis resistance is implicated in endometriosis,^[^ ^23^ ^]^ in the present study, we utilized western blot analysis to further investigate ferroptosis‐related marker proteins expression. Compared with EN tissues, EC tissues exhibited reduced expression of acyl‐CoA synthetase long‐chain family member 4 (ACSL4), a key promoter of ferroptosis, alongside significant upregulation of solute carrier family 7 member 11 (SLC7A11) and glutathione peroxidase 4 (GPX4), both critical inhibitors of ferroptosis (Figure S1A,B, Supporting Information). These findings provide molecular evidence supporting the occurrence of ferroptosis resistance in EMs. Several studies have demonstrated that lactate induces resistance to ferroptosis in tumors.^[^ ^24^, ^25^ ^]^ However, the effects of lactate and its mediated increase in histone lactylation on ferroptosis in EMs have not been reported. To explore the effect of histone lactylation on EMs, we stimulated EESCs with exogenous lactate or rotenone (a mitochondrial respiratory inhibitor) and measured the lactate concentration in EESCs. Both exogenous lactate and rotenone increased lactate levels in EESCs (**Figure** **2**A). Both exogenous lactate and rotenone also significantly increased global lactylation and H3K18la levels in EESCs (Figure 2B–D). To better optimize the concentration of lactate, we stimulated EESCs with varying concentrations of lactate. The results of the cell counting kit‐8 (CCK‐8) assay showed that a concentration of 10 mmol L^−1^ had the most significant effect on cell viability, and this effect was not due to pH interference (Figure S2A,B, Supporting Information). Moreover, we added erastin, an inducer of ferroptosis, to the culture media of EESCs for 24 h to establish a cellular ferroptosis model. The results of the CCK‐8 assay demonstrated that the viability of EESCs significantly increased after lactate stimulation following treatment with erastin (Figure 2E). In addition, lactate significantly increased GSH levels (Figure 2F) but decreased lipid peroxidation levels (Figure 2G–I) and intracellular Fe^2+^ levels (Figure 2J,K) upon erastin treatment. These findings suggest that lactate enhanced resistance to ferroptotic cell death. We further examined the effects of lactate on the expression of ferroptosis markers by qRT‐PCR and western blotting. The results indicated that lactate downregulated ACSL4 mRNA expression while upregulating SLC7A11 and GPX4 mRNA expression (Figure 2L–N). Consistently, lactate led to the downregulation of the ACSL4 protein and the upregulation of SLC7A11 and GPX4 protein expression (Figure 2O,P). Conversely, we further decreased the global intracellular histone lactylation level in EESCs through the use of glycolysis inhibitors, including the nonmetabolizable glucose analog 2‐DG and oxamate. The results revealed that both 2‐DG and oxamate decreased lactate levels in EESCs (**Figure** **3**A). Importantly, both glycolysis inhibitors led to a significant reduction in global lactylation and H3K18la levels in EESCs (Figure 3B–D). As anticipated, treatment with 2‐DG resulted in reduced viability of EESCs (Figure 3E) and decreased GSH levels (Figure 3F), while increasing intracellular lipid peroxidation (Figure 3G–I) and Fe^2^⁺ levels (Figure 3J,K). Notably, ferrostatin‐1, a ferroptosis inhibitor, partially mitigated the ferroptotic effects of 2‐DG in EESCs (Figure 3E–K). In addition, the effect of 2‐DG on ferroptosis markers was opposite to that of lactate (Figure 3L–P). Taken together, these results suggest that elevated histone lactylation levels contributed to resistance to ferroptosis in EMs and that targeting histone lactylation inhibition could be a potential therapeutic strategy for EMs.

### 2.3 Elevated Histone Lactylation Promotes METTL3 Upregulation in EESCs

**Figure 4:**
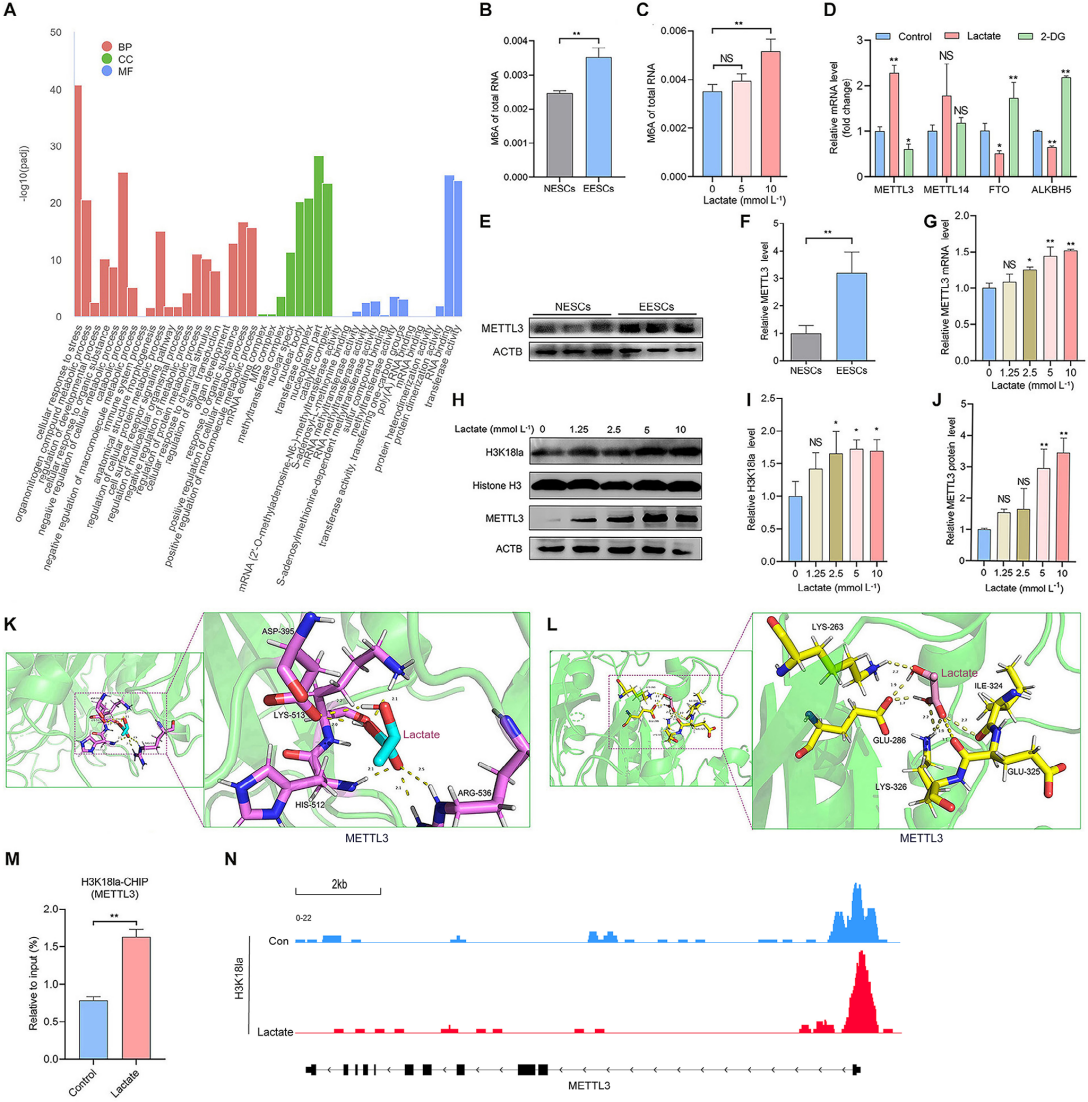
Elevated histone lactylation promotes METTL3 upregulation in EESCs. A) GO enrichment analysis of the genes targeted directly by H3K18la in EESCs in CUT&Tag assays. B) m6A levels were quantified in NESCs and EESCs. C) m6A levels were quantified in EESCs after stimulation with different concentrations of lactate (0, 5, and 10 mmol L^−1^) for 24 h. D) The mRNA levels of METTL3, METTL14, FTO, and ALKBH5 in EESCs were detected by qRT‐PCR after treatment with 10 mmol L^−1^ lactate or 20 mm 2‐DG for 24 h. E,F) Western blot analysis was used to detect the protein levels of METTL3 in NESCs and EESCs. G) METTL3 mRNA levels in EESCs were detected by qRT‐PCR after treatment with different concentrations of lactate (0, 1.25, 2.5, 5, and 10 mmol L^−1^) for 24 h. H–J) Western blot analysis was used to detect the protein levels of H3K18la and METTL3 in EESCs after stimulation with different concentrations of lactate (0, 1.25, 2.5, 5, and 10 mmol L^−1^) for 48 h. K) Molecular docking in the MOE was performed to predict the binding affinity between L‐lactic acid molecules and the MTD of METTL3. L) The MOE was used to predict the binding affinity between L‐lactic acid molecules and the ZFD of METTL3. M) The relative occupancy of H3K18la on the METTL3 promoter in EESCs treated with or without lactate (10 mmol L^−1^) for 24 h was analyzed by ChIP‒qPCR. N) Representative CUT&Tag assay images showing H3K18la binding at the METTL3 locus in EESCs, with or without lactate treatment. Error bars represent the mean ± SD. Two‐tailed Student's *t*‐test was used to compare the means of two groups; One‐way ANOVA was used to compare the means of more than two groups; NS no significant; ^*^ *p* < 0.05; ^**^ *p* < 0.01.

To investigate the mechanism by which enhanced histone lactylation contributes to ferroptosis resistance in EMs, we conducted Cleavage Under Targets and Tagmentation (CUT&Tag) assays of H3K18la to gain insights into the genes targeted directly by H3K18la in EESCs. Gene Ontology (GO) enrichment analysis revealed that these genes were associated primarily with biological process (BP), such as “regulation of cellular metabolic processes,” “regulation of developmental processes,”and “cellular response to stress” (**Figure** **4**A). Molecular function (MF) analysis further highlighted significant enrichment in “RNA binding” and “methyltransferase activity” (Figure 4A), suggesting that histone lactylation may regulate m6A modification. To delve deeper, we performed m6A quantification assays, which showed that m6A levels were significantly elevated in EESCs compared with NESCs (Figure 4B). Notably, lactate treatment further increased m6A levels in EESCs, indicating that histone lactylation modulates m6A modification (Figure 4C). qRT‐PCR analysis identified METTL3 was the most significantly altered m6A‐associated enzyme in response to lactate or 2‐DG treatment (Figure 4D). Western blotting confirmed METTL3 upregulation in EESCs compared with NESCs (Figure 4E,F). Additionally, qRT‐PCR results showed a positive correlation between METTL3 mRNA expression and lactate concentration (Figure 4G). Moreover, western blot analysis demonstrated that lactate was also capable of increasing METTL3 protein expression in line with H3K18la levels (Figure 4H–J). To understand this interaction at the molecular level, we utilized molecular docking in the Molecular Operating Environment (MOE) to predict the binding affinity between L‐lactic acid molecules and the methyltransferase domain (MTD) (Figure 4K) or zinc finger domain (ZFD) (Figure 4L) of METTL3. The results revealed the binding sites of L‐lactate molecules on the functional residues of METTL3, which altered the conformation of METTL3 by binding to lactate, thereby altering its ability to bind to other molecules (Figure 4K,L). Next, chromatin immunoprecipitation‐qPCR assay (ChIP‒qPCR) with anti‐H3K18la antibodies was performed to determine the regulatory effect of histone lactylation on METTL3 expression, revealing that H3K18la was enriched in the promoter region of METTL3 (Figure 4M). Notably, the CUT&Tag results highlighted indicated increased binding of H3K18la to METTL3 in EESCs following lactate stimulation (Figure 4N). Collectively, these results suggest that increased histone lactylation promotes METTL3 upregulation in EESCs.

### 2.4 Histone Lactylation Modulates Ferroptosis in EESCs by Regulating METTL3

**Figure 5:**
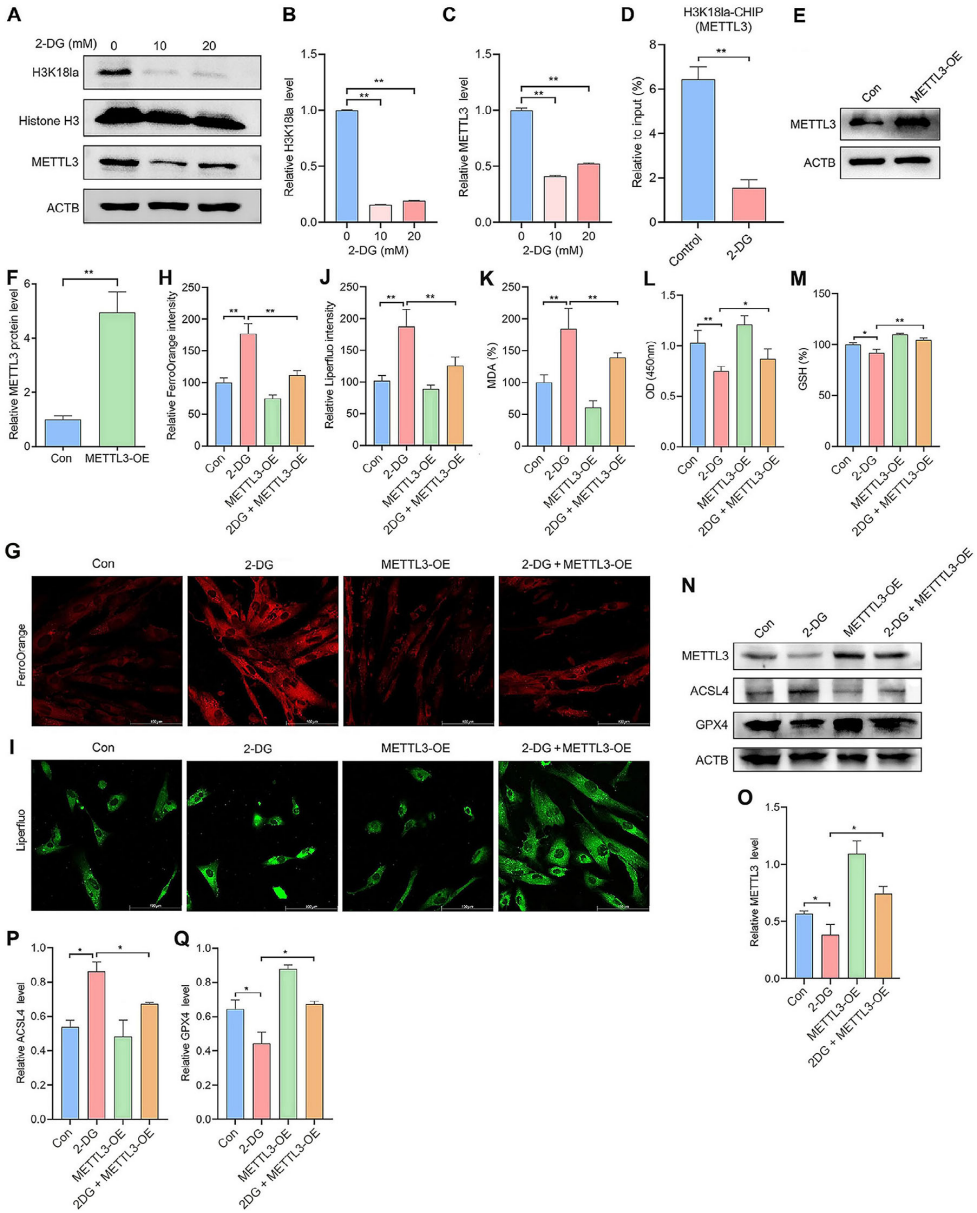
Histone lactylation modulates ferroptosis in EESCs by regulating METTL3. A–C) Western blot analysis was used to detect H3K18la and METTL3 protein levels in EESCs after treatment with different concentrations of 2‐DG (0, 10, and 20 mm) for 48 h. D) The relative occupancy of H3K18la at the METTL3 promoter in EESCs treated with or without 2‐DG (20 mm) for 24 h was analyzed by ChIP‒qPCR. E,F) Western blot analysis was used to detect METTL3 protein levels in EESCs after transfection with a METTL3 overexpression plasmid. G,H) Intracellular Fe^2+^ levels in the indicated groups were assessed by FerroOrange staining (scale bar = 100 µm). I–K) Intracellular lipid peroxidation levels were assessed by Liperfluo staining (scale bar = 100 µm) I,J) and MDA assays K) in the indicated groups. L) Cell viability was assessed by CCK‐8 assays in the indicated groups. M) GSH levels were measured by a GSH kit in the indicated groups. N–Q) Western blotting was used to detect the protein levels of ACSL4, GPX4, and METTL3 in the Con, 2‐DG, METTL3‐OE, and 2‐DG + METTL3‐OE groups. Error bars represent the mean ± SD. Two‐tailed Student's *t*‐test was used to compare the means of two groups; One‐way ANOVA was used to compare the means of more than two groups; ^*^ *p* < 0.05; ^**^ *p* < 0.01.

Several studies have reported that m6A modification is involved in the regulation of ferroptosis in tumors.^[^ ^26^ ^]^ To further validate the role of histone lactylation in modulating ferroptosis in EESCs through METTL3 regulation, western blot analysis was performed, which revealed that METTL3 protein expression was decreased in line with H3K18la levels after 2‐DG treatment (**Figure** **5**A–C). The results of the ChIP‒qPCR assays revealed a decrease in H3K18la enrichment in the METTL3 promoter region following 2‐DG treatment (Figure 5D). Furthermore, we transfected EESCs with a METTL3 overexpression plasmid (the overexpression efficiency of METTL3 is illustrated in Figure 5E,F) following the induction of decreased histone lactylation by 2‐DG. As expected, the elevated intracellular Fe^2+^ (Figure 5G,H) and lipid peroxidation (Figure 5I–K) in 2‐DG‐treated EESCs were reversed by METTL3 upregulation. In addition, we performed CCK‐8 assays and revealed that METTL3 overexpression markedly reversed the decrease in viability caused by 2‐DG (Figure 5L). The decrease in GSH levels induced by 2‐DG was also restored by METTL3 overexpression (Figure 5M). Importantly, METTL3 overexpression partially reversed the effects of 2‐DG, including the upregulation of ACSL4 and the downregulation of GPX4 in EESCs (Figure 5N–Q). Collectively, these findings suggest that histone lactylation modulated ferroptosis in EESCs by regulating METTL3 expression.

### 2.5 The HIF1A/HMOX1 Axis Serves as a Pivotal Signaling Pathway in METTL3‐Mediated Ferroptosis Resistance

**Figure 6:**
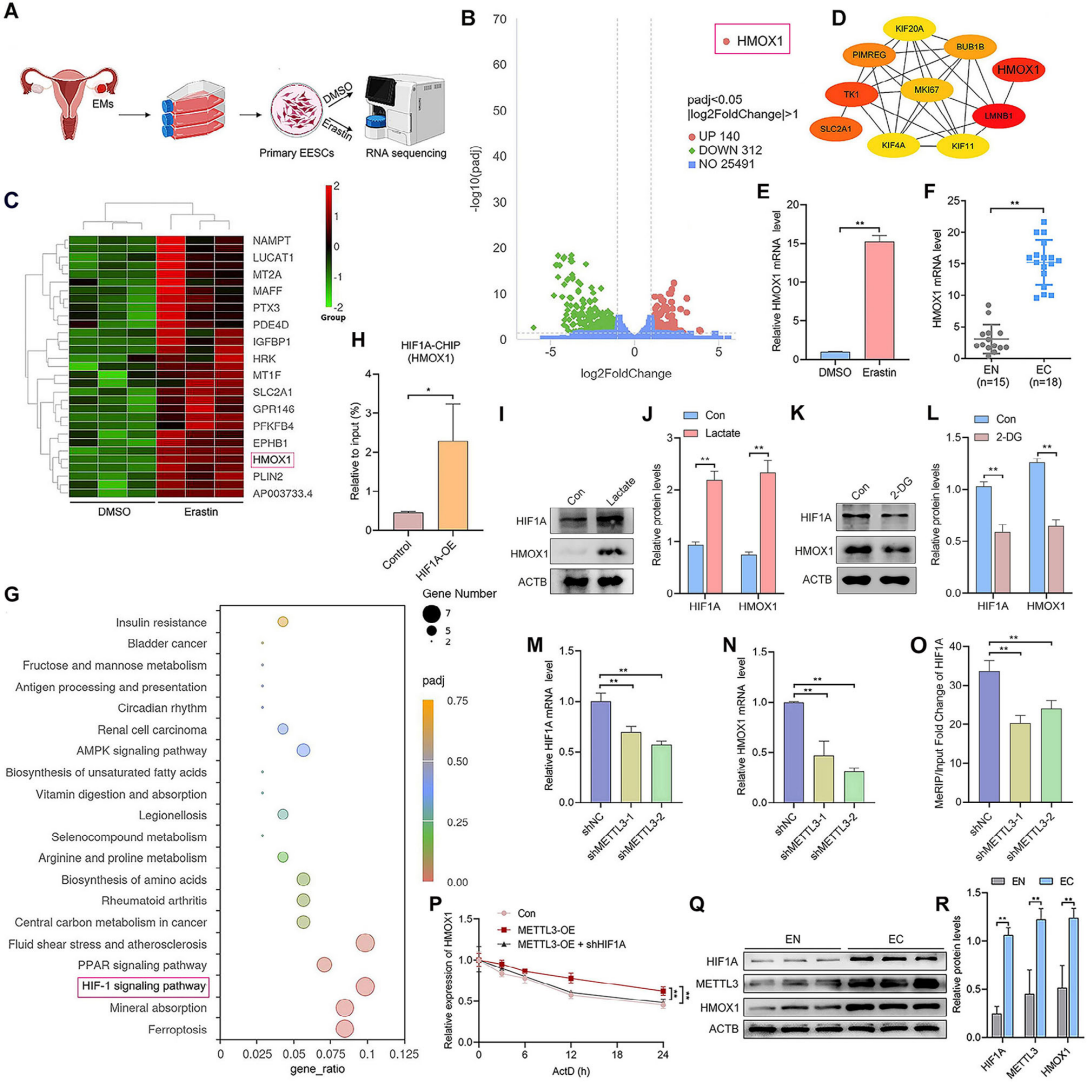
The HIF1A/HMOX1 axis serves as a pivotal signaling pathway in METTL3‐mediated ferroptosis resistance. A) Schematic representation of the whole transcriptome gene sequencing of EESCs. B) Differentially expressed transcripts (erastin vs DMSO; log2‐fold change >1, padj < 0.05) are shown in the volcano plot (*n* = 3). Red dots indicate upregulated genes, and green dots indicate downregulated genes. C) Heatmap of upregulated genes, with low expression shown in green and high expression in red. D) Among the differentially expressed genes, the Cytoscape 3.10 software cytoHubba plugin was applied to the top 10 priority genes in the network based on the STRESS method. Nodes represent genes, color shades indicate weighted degree scores, and black lines represent interactions. E,F) qRT‐PCR was used to detect the mRNA levels of HMOX1 in EESCs after treatment with 10 µm erastin for 24 h E) and in EN (*n* = 15) and EC (*n* = 18) tissues F). G) Bubble chart of KEGG pathway enrichment analysis of the DEGs. H) The relative occupancy of HIF1A at the HMOX1 promoter in EESCs was analyzed by ChIP‒qPCR after transfection with HIF1A overexpression plasmids or control plasmids for 24 h. I,J) Western blot analysis was performed to detect the protein expression levels of HIF1A and HMOX1 in EESCs after lactate treatment. K,L) Western blot analysis was performed to detect the protein expression levels of HIF1A and HMOX1 in EESCs after 2‐DG treatment. M,N) qRT‐PCR was used to determine HIF1A M) and HMOX1 N) mRNA levels in EESCs transfected with METTL3 knockdown plasmids for 24 h. O) After transfection of EESCs with METTL3 knockdown plasmids for 24 h, m6A‐enriched HIF1A mRNA was quantified by MeRIP‒qPCR. P) mRNA decay analysis of HMOX1 in EESCs was performed following METTL3 overexpression, with or without concurrent HIF1A knockdown, at the indicated time points after treated with actinomycin D (5 µg mL^−1^). Q,R) Western blot analysis was performed to detect the protein expression levels of METTL3, HIF1A, and HMOX1 in EN (*n* = 9) and EC (*n* = 9) tissues. Error bars represent the mean ± SD. Two‐tailed Student's *t*‐test was used to compare the means of two groups; One‐way ANOVA was used to compare the means of more than two groups; ^*^ *p* < 0.05; ^**^ *p* < 0.01.

To explore the mechanism of METTL3 in ferroptosis resistance, primary EESCs were treated with dimethyl sulfoxide (DMSO) or 10 µm erastin, and total RNA was extracted and subjected to transcriptome‐wide gene sequencing. The flowchart is presented in **Figure** **6**A. The volcano plot revealed 452 differentially expressed genes (DEGs), 140 of which were upregulated and 312 of which were downregulated (log2‐fold change > 1, padj < 0.05), relative to the DMSO group. Among the upregulated genes, the antioxidant factor HMOX1 was the most significantly upregulated (Figure 6B). The heatmap analysis further confirmed that HMOX1 was one of the most significantly upregulated genes (Figure 6C). Using the STRESS method with the Cytoscape 3.10 software cytoHubba plugin, we identified the top 10 genes with the highest priority, with HMOX1 ranking the highest (Figure 6D). qRT‐PCR experiments confirmed the increase in HMOX1 mRNA in EESCs following erastin treatment (Figure 6E). Additionally, elevated HMOX1 expression was also detected in ferroptosis‐resistant EC tissues compared to EN tissues (Figure 6F). On the basis of these reports, we hypothesize that HMOX1 plays a crucial role in ferroptosis in EMs. Moreover, kyoto encyclopedia of genes and genomes (KEGG) enrichment analysis of the DEGs revealed the greatest enrichment in the “HIF‐1 signaling pathway” (Figure 6G). In the present study, we performed ChIP‒qPCR and confirmed that HIF‐1A could bind to the HMOX1 promoter (Figure 6H). Western blot analysis demonstrated that lactate treatment upregulated HIF1A and HMOX1 protein levels in EESCs (Figure 6I,J). In contrast, 2‐DG treatment suppressed the expression of both proteins in EESCs (Figure 6K,L). These results indicate that HMOX1 was a downstream target gene of HIF‐1A and that the HIF1A/HMOX1 signaling pathway played an important role in ferroptosis in EMs. Bioinformatics analysis revealed that HIF1A could be a potential downstream target of METTL3 (Figure S3A,B, Supporting Information). Upon transfecting EESCs with METTL3 knockdown plasmids (shMETTL3‐1 and shMETTL3‐2), qRT‐PCR revealed decreased mRNA levels of HMOX1 and HIF1A (Figure 6M,N). Methylated RNA Immunoprecipitation‒qPCR (MeRIP‐qPCR) further verified that METTL3 knockdown significantly reduced the enrichment of HIF‐1A mRNA via m6A‐specific antibodies (Figure 6O). After treating EESCs with actinomycin D (ActD) to block transcription, we found that the level of HMOX1 mRNA was shown to be highly stable under the condition of METTL3 overexpression, whereas HIF1A knockdown significantly decreased the half‐life of HMOX1 mRNA following METTL3 overexpression (Figure 6P). Finally, western blot assays indicated that the protein levels of METTL3, HIF1A, and HMOX1 were upregulated in EC tissues compared with EN tissues (Figure 6Q,R). Collectively, these results suggest that HIF1A/HMOX1 may serve as the key signaling pathway in METTL3‐mediated ferroptosis resistance.

### 2.6 METTL3 Confers Ferroptosis Resistance via Modulation of the HIF1A/HMOX1 Signaling Pathway

**Figure 7:**
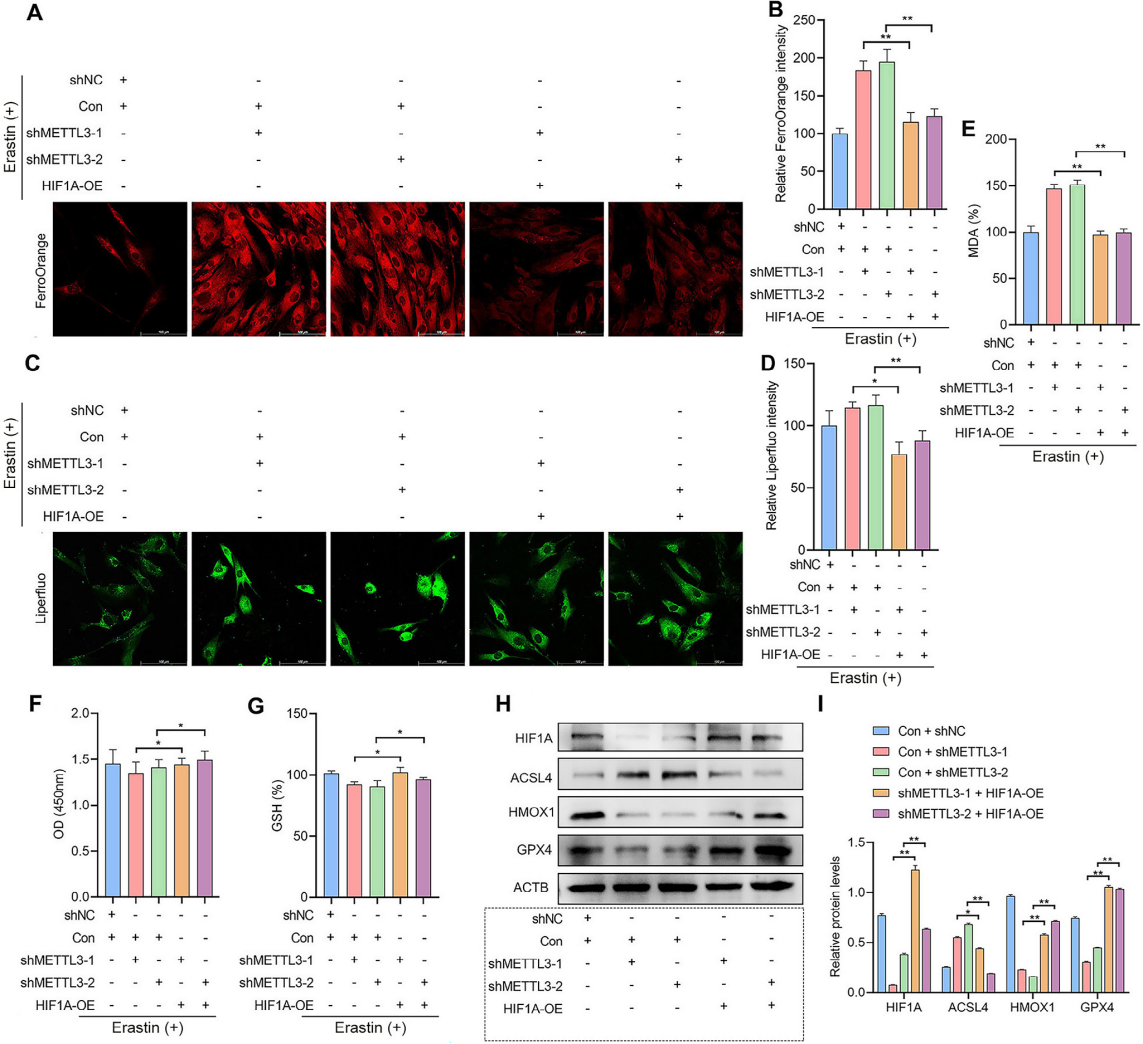
METTL3 knockdown promotes ferroptosis via modulation of the HIF1A/HMOX1 pathway. A–G) A ferroptosis model was established by stimulating EESCs with 10 µm erastin. EESCs were then transfected with different plasmids (shNC + Con, shMETTL3‐1 + Con, shMETTL3‐2 + Con, shMETTL3‐1 + HIF1A‐OE, and shMETTL3‐2 + HIF1A‐OE) for 24 h. A) Intracellular Fe^2+^ levels in EESCs were assessed by FerroOrange staining (scale bar = 100 µm). B) Statistical analysis of the intracellular Fe^2+^ levels. C) Intracellular lipid peroxidation levels in EESCs were assessed by Liperfluo staining (scale bar = 100 µm). D) Statistical analysis of changes in intracellular lipid peroxidation levels. E) MDA levels in EESCs were measured using MDA kits. F) Cell viability was measured by the CCK‐8 assay. G) GSH levels were measured using a GSH kit. H) Western blotting of samples from cells treated with METTL3 knockdown plasmids (shMETTL3‐1 and shMETTL3‐2) with or without HIF1A overexpression plasmids (HIF1A‐OE) for 48 h. I) Statistical analysis of protein expression.

To determine the role of METTL3 in mediating ferroptosis resistance in EESCs through the HIF1A/HMOX1 pathway, we first established an in vitro ferroptosis model by stimulating EESCs with 10 µm erastin. METTL3 knockdown resulted in increased intracellular Fe^2+^ (**Figure** **7**A,B) and elevated lipid peroxidation (Figure 7C–E). Conversely, overexpression of HIF1A partially reversed the effects induced by METTL3 inhibition (Figure 7A–E). Furthermore, the results of the CCK‐8 assay demonstrated that METTL3 knockdown reduced cell viability, whereas HIF1A overexpression restored cell viability (Figure 7F). Similar results were obtained in the GSH assays (Figure 7G). Additionally, the effects of METTL3 knockdown and HIF1A overexpression on the expression of ferroptosis‐related proteins were evaluated via western blot analysis. These results showed that METTL3 knockdown resulted in increased ACSL4 protein expression and decreased HIF1A, HMOX1, and GPX4 protein expression. The overexpression of HIF1A partially reversed the effects of METTL3 knockdown on ACSL4, HIF1A, HMOX1, and GPX4 expression (Figure 7H,I). In conclusion, these findings indicate that HIF1A overexpression mitigated the ferroptosis facilitated by METTL3 knockdown.

**Figure 8:**
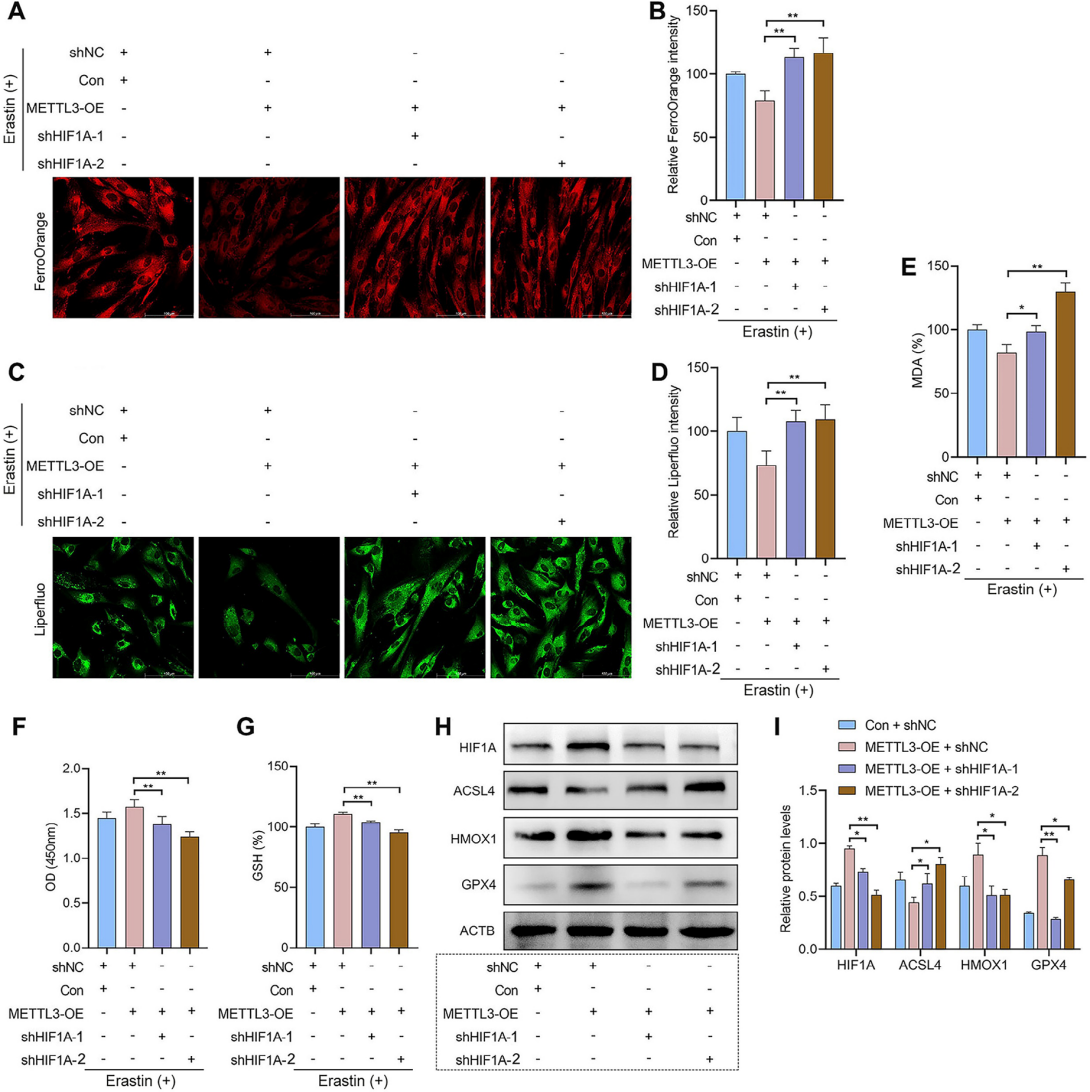
METTL3 overexpression confers ferroptosis resistance via modulation of the HIF1A/HMOX1 signaling pathway. A–G) A ferroptosis model was established by stimulating EESCs with 10 µm erastin. EESCs were then transfected with different plasmids (shNC + Con, METTL3‐OE + shNC, METTL3‐OE + shHIF1A‐1, and METTL3‐OE + shHIF1A‐2) for 24 h. A) Intracellular Fe^2+^ levels in EESCs were assessed by FerroOrange staining (scale bar = 100 µm). B) Statistical analysis of the intracellular Fe^2+^ levels. C) Intracellular lipid peroxidation levels in EESCs were assessed by Liperfluo staining (scale bar = 100 µm). D) Statistical analysis of changes in intracellular lipid peroxidation levels. E) MDA levels in EESCs were measured using MDA kits. F) Cell viability was measured by the CCK‐8 assay. G) GSH levels were measured using a GSH kit. H) Western blotting of samples from cells treated with a METTL3 overexpression plasmid (METTL3‐OE) with or without HIF1A knockdown plasmids (shHIF1A‐1 and shHIF1A‐2) for 48 h. I) Statistical analysis of protein expression. Error bars represent the mean ± SD. One‐way ANOVA was used to compare the means of more than two groups; ^*^ *p* < 0.05; ^**^ *p* < 0.01.

In contrast, HIF1A knockdown partially reversed the effects of METTL3 overexpression on intracellular Fe^2+^ (**Figure** **8**A,B), lipid peroxidation (Figure 8C–E), cell viability (Figure 8F), and GSH levels (Figure 8G) during ferroptosis in EESCs. In addition, METTL3 overexpression resulted in the downregulation of ACSL4 protein expression and the upregulation of HIF1A, HMOX1, and GPX4 protein expression. HIF1A knockdown partially rescued the effects of METTL3 overexpression on those proteins expression (Figure 8H,I). These results suggest that HIF1A knockdown counteracted the ferroptosis resistance induced by METTL3 overexpression. In conclusion, our experiment demonstrates that METTL3 mediated ferroptosis resistance in EESCs by modulating the HIF1A/HMOX1 signaling pathway.

### 2.7 The Combination of 2‐DG and Erastin Alleviates Endometriosis in a Mouse Model

**Figure 9:**
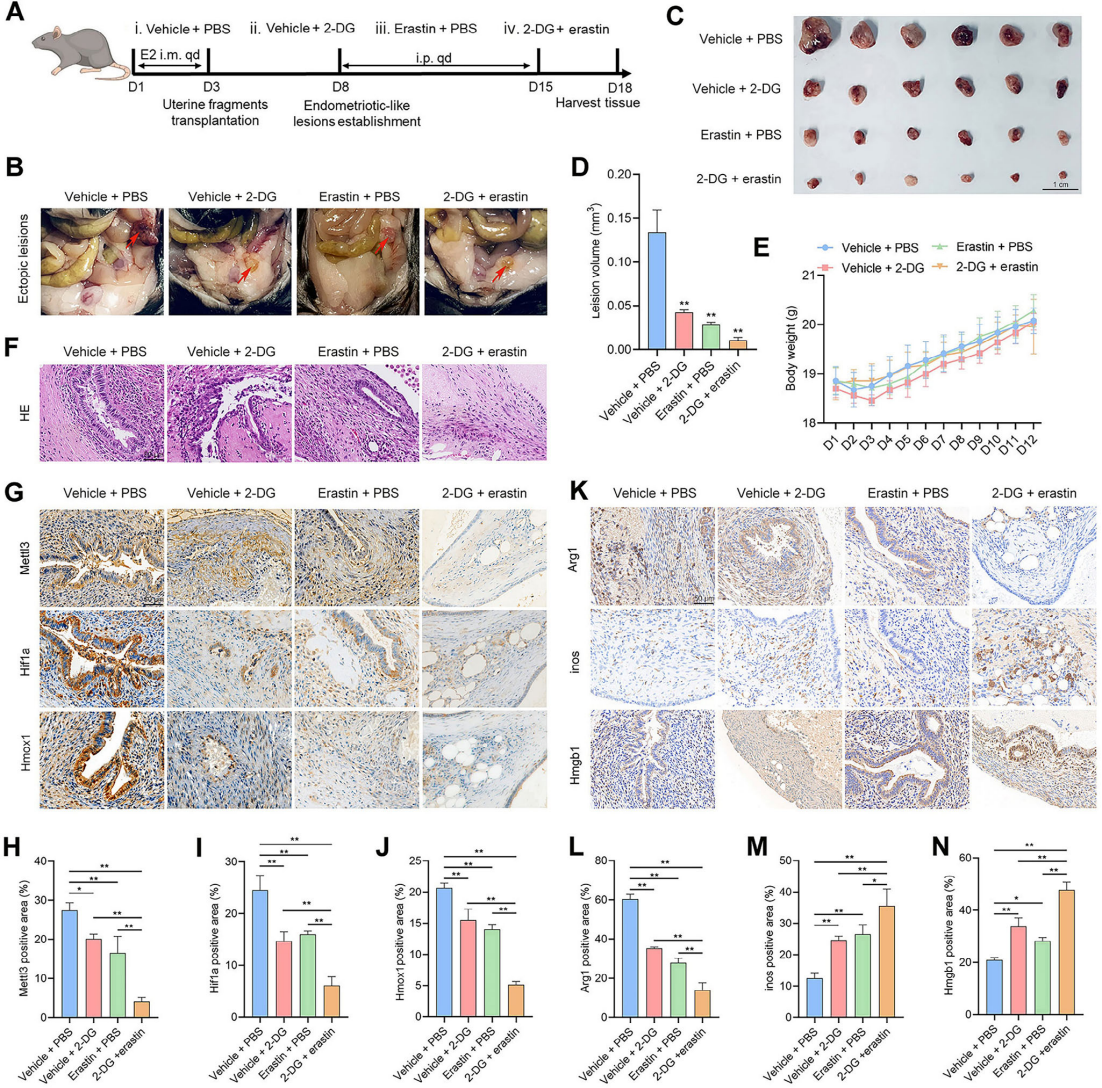
The combination of 2‐DG and erastin alleviated endometriosis in a mouse model. A) Flowchart illustrating the establishment of the mouse EMs therapeutic model. B,C) Representative images showing visible lesions in the peritoneal cavity of EMs mice after treatment with vehicle + PBS (*n* = 6), vehicle + 2‐DG (*n* = 6), erastin + PBS (*n* = 6), or 2‐DG + erastin (*n* = 6) for 7 days. Erastin was administered at a dosage of 20 mg kg^−1^ d^−1^, and 2‐DG was administered at 100 mg kg^−1^ d^−1^. D) Comparison of the sizes of the ectopic lesions in the indicated groups. E) Body weight measurements of animals in the indicated groups. F) HE staining depicting the glandular and stromal structures of ectopic lesions in the indicated groups (scale bar = 50 µm). G–J) IHC analyses of Mettl3, Hif1a, and Hmox1 in endometriotic lesions (scale bar = 50 µm). K–N) IHC analyses of Arginase 1 (Arg1), inducible nitric oxide synthase (inos), and high mobility group box 1 (Hmgb1) in endometriotic lesions (scale bar = 50 µm). Error bars represent the mean ± SD. One‐way ANOVA was used to compare the means of more than two groups; ^**^ *p* < 0.01.

To further assess whether combined treatment with 2‐DG and erastin has a therapeutic effect on EMs, we established an EMs mouse model, as depicted in the schematic diagram (**Figure** **9**A). After 5 days of ectopic lesion formation, EMs mice were divided into four groups: vehicle + phosphate‐buffered saline (PBS), vehicle + 2‐DG, erastin + PBS, and 2‐DG + erastin. Ectopic lesions were then collected, revealing that, compared with treatment with 2‐DG or erastin alone, the combination of 2‐DG and erastin significantly reduced the ectopic lesion volume (Figure 9B–D). Moreover, there were no statistically significant differences in mouse body weight between the groups (Figure 9E). Hematoxylin and eosin (HE) staining revealed that the glandular and stromal structures of the ectopic lesions were significantly disrupted by the combination of 2‐DG and erastin (Figure 9F). Additionally, the immunohistochemical (IHC) staining results revealed a more pronounced decrease in the protein levels of Mettl3, Hif1a, and Hmox1 in response to the combination of 2‐DG and erastin (Figure 9G–J).

**Figure 10:**
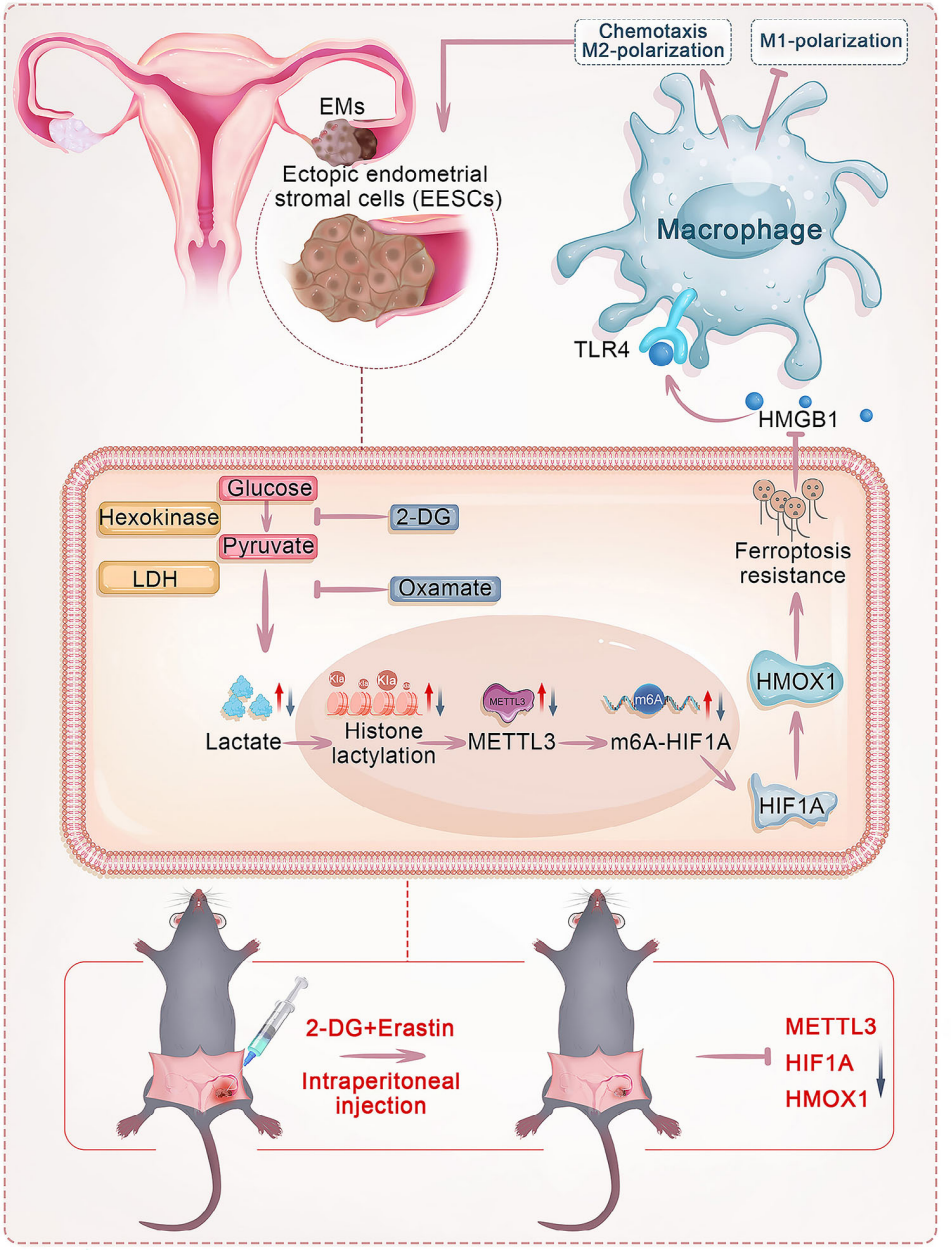
Schematic illustration showing that elevated histone lactylation facilitates ferroptosis resistance in EMs through the METTL3‐regulated HIF1A/HMOX1 signaling pathway. In addition, histone lactylation‐mediated ferroptosis resistance in EESCs promotes macrophage chemotaxis and M2 polarization, inhibits M1 polarization.

Our prior studies indicate that macrophages in EMs predominantly exhibit an M2 phenotype, which facilitates disease onset and progression, whereas M1 macrophages play an inhibitory role.^[^ ^27^, ^28^ ^]^ Previous studies have shown that cellular ferroptosis triggers the release of damage‐associated molecular patterns (DAMPs), with extracellular HMGB1 acting as a DAMP through TLR4 to recruit macrophages and shift M2 macrophages to the M1 phenotype.^[^ ^29^, ^30^ ^]^ Thus, we hypothesize that alterations of ferroptosis status in EESCs could affect macrophage polarization. In this study, we found that elevated histone lactylation in EESCs suppressed HMGB1 expression and secretion (Figure S4A,B, Supporting Information), which subsequently enhanced macrophage chemotaxis (Figure S4D–F, Supporting Information) and promoted M2 polarization, and inhibited M1 polarization (Figure S4I–K,N–P, Supporting Information). Conversely, reduced histone lactylation elevated HMGB1 expression and secretion (Figure S4A,C, Supporting Information), which led to decreased macrophage chemotaxis (Figure S4D,G,H, Supporting Information), favored M1 polarization, and inhibited M2 polarization (Figure S4I,L–P, Supporting Information). Furthermore, in vivo IHC revealed a substantial reduction in proteins related to M2 macrophage polarization and an augmentation of the M1 phenotype upon administration of a combination of 2‐DG and erastin compared with treatment with 2‐DG or erastin alone (Figure 9K–N).The positive and negative controls for IHC are shown in Figure S5 (Supporting Information).

Collectively, these findings suggest that the combination of 2‐DG and erastin enhanced therapeutic efficacy in EMs mice by significantly reducing the protein levels of Mettl3, Hif1a, and Hmox1, inhibiting M2 macrophages, and promoting the M1 macrophage phenotype **(Figure** 10).

## 3 Discussion

Our previous study highlighted the occurrence of ferroptosis resistance in EMs.^[^ ^23^ ^]^ Some studies have suggested that EESCs develop resistance to ferroptosis because of their prolonged exposure and tolerance to high levels of iron and reactive oxygen species.^[^ ^9^ ^]^ However, the specific mechanism underlying this phenomenon remains elusive. Recent reports have identified lactate as a major source of histone lactylation, a newly defined PTM.^[^ ^15^, ^31^ ^]^ This modification, particularly H3K18la, has been found to regulate various biological processes, including tumorigenesis, tumor progression, immune escape, and metabolic reprogramming of cancer cells.^[^ ^32^, ^33^ ^]^ In the present study, we observed a significant increase in the histone lactylation level in EMs. Furthermore, we confirmed that elevated lactylation levels mediate ferroptosis resistance.

m6A is the most common posttranscriptional RNA modification in mammals.^[^ ^34^, ^35^ ^]^ METTL3 functions as the catalytic core of RNA methylation by acting as a m6A methyltransferase.^[^ ^36^ ^]^ Recent studies have highlighted the role of histone lactylation in mediating m6A modification to regulate tumorigenesis.^[^ ^37^ ^]^ Interestingly, several studies have suggested the involvement of m6A in the regulation of ferroptosis. Yang et al. showed that hypoxia‐inducible lncRNA CBSLR modulates ferroptosis through m6A‐YTHDF2‐dependent modulation of CBS in gastric cancer.^[^ ^38^ ^]^ Additionally, Zhang et al. reported that METTL3‐mediated m6A exacerbates ferroptosis via IGF2BP2‐dependent mitochondrial metabolic reprogramming in sepsis‐induced acute lung injury.^[^ ^39^ ^]^ However, whether histone lactylation can influence ferroptosis resistance in EMs by modulating m6A remains unexplored. In this study, we observed that elevated histone lactylation correlates with increased m6A modification levels in EMs, with a significant upregulation of METTL3 expression among various m6A‐related enzymes. ChIP‐qPCR analysis and CUT&tag assay further demonstrated the association between H3K18la and METTL3. Notably, METTL3 expression decreased upon reduction of histone lactylation levels, which was accompanied by a decrease in H3K18la enrichment at the METTL3 promoter region. Moreover, the reduction in histone lactylation‐mediated inhibition of ferroptosis resistance in EESCs was reversed by METTL3 overexpression. Mechanistically, METTL3 conferred ferroptosis resistance in EESCs by regulating the HIF1A/HMOX1 signaling pathway. Collectively, our results demonstrate that increased histone lactylation contributed to ferroptosis resistance in EMs through the HIF1A/HMOX1 signaling pathway, which was regulated by METTL3. Previous studies have demonstrated extensive M2 macrophage infiltration in the endometriotic pelvis, with these cells playing a critical role in the progression of EMs by promoting cell proliferation, invasion, and neovascularization.^[^ ^40^, ^41^ ^]^ In this study, we found that ferroptosis status alterations in EESCs regulated macrophage chemotaxis and polarization through HMGB1. Taken together, these findings indicate that a 2‐DG‐induced decrease in histone lactylation could not only promote ferroptosis in EESCs to achieve therapeutic effects but also inhibit macrophage chemotaxis and M2 polarization, thereby better inhibiting the occurrence and development of EMs and providing a new direction for the treatment of EMs. Furthermore, we established a mouse model of EMs and reported that 2‐DG combined with erastin significantly reduced the size of ectopic lesions compared with 2‐DG or erastin alone, suggesting that the combination of 2‐DG and erastin had better effects and greater therapeutic potential, providing a new theoretical basis for the subsequent treatment of EMs. We also found that the combination of 2‐DG and erastin resulted in a more significant decrease in the protein levels of Mettl3, Hif1a, and Hmox1, compared to either 2‐DG or erastin alone, suggesting that to some extent, histone lactylation regulated ferroptosis and ferroptosis resistance in EMs through the METTL3/HIF1A/HMOX1 axis. Furthermore, in vivo experiments demonstrated a more pronounced reduction in M2 macrophage polarization and a significantly greater increase in the M1 phenotype in the combined 2‐DG and erastin treatment group compared to either treatment alone.

In conclusion, our findings show that histone lactylation orchestrated ferroptosis resistance in EMs through the METTL3‐regulated HIF1A/HMOX1 signaling pathway. In vivo, combined treatment with 2‐DG and erastin was more effective in treating ectopic lesions in EMs. Our findings suggest that histone lactylation may serve as a biomarker for the development of EMs. Combined therapy that inhibits histone lactylation and induces ferroptosis may be a new strategy for the treatment of EMs.

## 4 Experimental Section

### Clinical Specimens

Twenty‐one patients who were diagnosed with endometriosis through laparoscopy and histological analysis at the Second Affiliated Hospital of Harbin Medical University from October 2021 to August 2023 were included in the study. Normal endometrial tissues were collected from 15 patients who underwent benign gynecological procedures, such as uterine prolapse and benign cervical lesions, and who did not exhibit clinical symptoms or signs of endometriosis or adenomyosis (**Table** **1**). Tissue samples were obtained from patients during the phase of increased cell proliferation in their menstrual cycle. Ethical approval for this study was obtained from the Ethical Committee of the Second Affiliated Hospital of Harbin Medical University, and all participants provided informed written consent.

**Table 1:** Clinical characteristics of female with and without (control) endometriosis.

	Control [*n* = 15]^a)^	Endometriosis [*n* = 21]	*P* value
Age [year]	40.97 ± 6.06	37.14 ± 7.07	0.099
BMI [kg m^−2^]	23.37 ± 0.4	23.44 ± 0.56	0.923
CA125 level [U mL^−1^	18.68 ± 12.46	64.42 + 80.15	0.036
Menstrual average cycle [day]	29.35 ± 1.25	29.83 ± 2.25	0.458
Menstrual duration [day]	5.34 ± 1.28	5.88 ± 1.36	0.237
r‐AFS stage			
Stage I [minimal]		0/21	
Stage II [mild]		Apr‐21	
Stage III [moderate]		Oct‐21	
Stage IV [severe]		Jul‐21	

^a)^ Statistical analysis was performed using Student's *t*‐test. Data are mean ± SD. AFS, American Fertility Society.

### Cell Culture

Primary EESCs were isolated and cultured following previously described methods.^[^ ^28^ ^]^ Briefly, the tissues were cut into ≈1 mm^3^ pieces and digested with 4% type IV collagenase (Sigma, USA) in a shaking water bath at 37 °C for 60 min. The dispersed endometrial cells were then separated by 100 and 400 stainless steel mesh sieves. The filtrate was subsequently centrifuged at 1000 rpm for 3 min and washed with Dulbecco's modified Eagle's medium (DMEM) three times to remove erythrocytes. The remaining cells were resuspended and cultured in DMEM supplemented with 15% fetal bovine serum (FBS; Gibco, USA) and 1% penicillin‒streptomycin (Gibco, USA) at 37 °C with 5% CO_2_. After 12 h, the culture medium was replaced. EESCs were identified by IF staining for vimentin (+) and cytokeratin 7 (−) and were used for subsequent in vitro experiments in triplicate.^[^ ^27^ ^]^

The acute monocytic leukemia cell line (THP‐1) was purchased from the American Type Culture Collection (ATCC, USA) and cultured in Roswell Park Memorial Institute 1640 (RPMI‐1640; Gibco, USA) medium supplemented with 10% FBS and 1% penicillin‒streptomycin (Gibco, USA). THP‐1 cells were subsequently treated with phorbol‐12‐myristate‐13‐acetate (PMA; 200 nm; Sigma, USA) for 48 h to induce macrophages.

### IHC Staining

IHC staining assays were conducted following conventional protocols. The processed samples were sectioned into 4 µm slices, which were then incubated overnight and immersed in xylene and ethanol for deparaffinization. Next, the sections were subjected to a 16 h incubation with primary antibodies (as listed in **Table** **2**) at 4 °C, followed by incubation with secondary antibodies for 30 min at room temperature. Positive staining was developed using a diaminobenzidine (DAB) staining kit (CWBIO, China) and counterstained with hematoxylin to detect horseradish peroxidase (HRP) activity. Finally, cover slips were applied to the slides.

**Table 2:** Details of antibody used in experiments.

Antigen^a)^	Catalog number	Concentration [µg mL^−1^]	Source	Species
WB				
ACTB	60008‐1‐Ig	0.05	Proteintech	Mouse
METTL3	67733‐1‐Ig	0.2	Proteintech	Mouse
HIF1A	20960‐1‐AP	1.5	Proteintech	Rabbit
HMOX1	66743‐1‐Ig	1	Proteintech	Mouse
Histone H3	PTM6600	1	PTM BIO	Rabbit
Pan Kla	PTM1401RM	1	PTM BIO	Rabbit
H3K18la	PTM1427RM	2	PTM BIO	Rabbit
ACSL4	22401‐1‐AP	0.75	Proteintech	Rabbit
SLC7A11	26864‐1‐AP	0.5	Proteintech	Rabbit
GPX4	14432‐1‐AP	0.5	Proteintech	Rabbit
TLR4	19811‐1‐AP	1	Proteintech	Rabbit
ARG1	66129‐1‐Ig	0.3	Proteintech	Mouse
iNOS	18985‐1‐AP	1.5	Proteintech	Rabbit
IF				
Pan Kla	PTM1401RM	10	PTM BIO	Rabbit
H3K18la	PTM‐1406RM	10	PTM BIO	Rabbit
GLUT1	21829‐1‐AP	0.5	Proteintech	Rabbit
LDHA	19987‐1‐AP	1.3	Proteintech	Rabbit
ENO1	11204‐1‐AP	1.2	Proteintech	Rabbit
IHC				
Mettl3	67733‐1‐Ig	1	Proteintech	Mouse
Hif1a	20960‐1‐AP	3	Proteintech	Rabbit
Hmox1	66743‐1‐Ig	1	Proteintech	Mouse
Arg1	66129‐1‐Ig	0.75	Proteintech	Mouse
inos	18985‐1‐AP	6	Proteintech	Rabbit

^a)^ *ACSL4* acyl‐CoA synthetase long‐chain family member 4, *ACTB* Beta‐actin, *SLC7A11* solute carrier family 7 member 11, *GPX4* glutathione peroxidase 4, *METTL3* methyltransferase like 3, *HIF1A* hypoxia‐inducible factor 1 alpha, *HMOX1* heme oxygenase 1, *TLR4* Toll‐like receptor 4, *ARG1* Arginase 1, *iNOS* Inducible NO synthase, *GLUT1* Glucose transporter 1, *LDHA* Lactate dehydrogenase A, *ENO1* alpha‐enolase.

### RNA Extraction and Quantitative Real‐Time PCR

Total RNA was extracted from frozen tissues or cells with TRIzol (Invitrogen, USA) following the manufacturer's protocol. First‐strand cDNA was synthesized from 500 ng of total RNA using a reverse transcription kit (Toyobo Co., Japan). The synthesized cDNA served as a template for quantitative real‐time PCR (qRT‐PCR), which was performed using SYBR Green PCR Master Mix (Bio‐Rad, USA) and an Applied Biosystems 7300 Real‐Time PCR System. The primers utilized in this study are listed in **Table** **3**. The expression levels were normalized to beta‐actin (ACTB) RNA expression. The mean relative gene expression was determined, and differences were calculated by the 2^−ΔΔCt^ method.

**Table 3:** List of primers for RT‐PCR.

Genes^a)^	Primers 5’‐3’
Human ACTB‐F	AGCGAGCATCCCCCAAAGTT
Human ACTB‐R	AGGGCACGAAGGCTCATCATT
Human ACSL4‐F	TTCGATTAAGCCCAGAGCCA
Human ACSL4‐R	GAGGTAATGGTTCCTCAGCTCCT
Human GPX4‐F	GGCAGACCCGAAAATCCAG
Human GPX4‐R	GTTTATTCCCACAAGGTAGCCA
Human SLC7A11‐F	CCCTTTGCTCTCATACCCATC
Human SLC7A11‐F	GACTTTCCTCTTCAGCTGCACTT
Human METTL3‐F	CAAGCTGCACTTCAGACGAA CACTTCAGACGAA
Human METTL3‐R	GCTTGGCGTGTGGTCTTT
Human METTL14‐F	GCCGTGTTAAATAGCAAAG GAGGAGGTAGTGGTTA
Human METTL14‐R	TGGAGCAGAGGTATCATAG
Human FTO‐F	GAAGGCTAATGAGGATGCTGTG
Human FTO‐R	GTTGTATGCTGCTCTGCTCTT
Human ALKBH5‐F	TCATCAACGACTACCAGCC
Human ALKBH5‐R	GAAGGACACGGACACGAT
Human HIF1A‐F	TGAGTTCGCATCTTGATAAGGC
Human HIF1A‐R	ACAAAACCATCCAAGGCTTTCA
Human HMOX1‐F	ACTGCGTTCCTGCTCAACAT
Human HMOX1‐R	CAGCATGCCTGCATTCACAT

^a)^ *ACSL4* acyl‐CoA synthetase long‐chain family member 4, *ACTB* Beta‐actin, *SLC7A11* solute carrier family 7 member 11, *GPX4* glutathione peroxidase 4, *METTL3* methyltransferase like 3, *HIF1A* hypoxia‐inducible factor 1 alpha, *HMOX1* heme oxygenase 1, *METTL14* methyltransferase like 14, *FTO* fat mass and obesity‐associated protein, *ALKBH5* alkB homolog 5.

### Western Blotting

Protein from cells was extracted with RIPA lysis buffer supplemented with protease inhibitor cocktail (Thermo Fisher Scientific, USA). The protein concentration was measured with a bicinchoninic acid protein assay kit (Beyotime Biotechnology, China). Equal amounts of proteins were loaded onto 10% or 12.5% sodium dodecyl sulfate–polyacrylamide gel electrophoresis (Epizyme, China) and subsequently transferred onto polyvinylidene fluoride membranes (Millipore, USA). The membranes were blocked with a 5% skim milk powder solution for 2 h, followed by overnight incubation with primary antibodies (as listed in Table 2) at 4 °C. Afterward, the membranes were incubated with secondary antibodies for 2 h at room temperature. The blots were visualized with an enhanced chemiluminescence (ECL) kit (Thermo Fisher Scientific, USA) and quantitated with Quantity One software (Bio‐Rad, USA). Relative target protein levels were determined as ratios of their gray value to that of ACTB, which served as an internal reference.

### Cell Transfection

METTL3 knockdown plasmids (shMETTL3‐1 and shMETTL3‐2), HIF1A knockdown plasmids (shHIF1A‐1, and shHIF1A‐2), a negative control plasmid (shNC), a METTL3 overexpression plasmid (METTL3‐OE), an HIF1A overexpression plasmid (HIF1A‐OE), and an overexpression blank control plasmid (Con) were designed and synthesized by Gene Pharma (Hanbio Biotechnology, China). EESCs were seeded in 96‐ or 6‐well plates and transfected with knockdown or overexpression plasmids at ≈70% confluence. The DNA and P3000 (Invitrogen, USA) were diluted with Opti‐MEM (Gibco, USA). Simultaneously, Lipo3000 (Invitrogen, USA) was diluted with Opti‐MEM. The two solutions were mixed and incubated for 20 min at room temperature. The mixture was added to the plates, and the supernatants were replaced after 4–6 h.

### CCK‐8 Assay

EESCs were seeded in 96‐well plates. Cell viability was assessed by the CCK‐8 assay according to the manufacturer's instructions. Briefly, the working solution containing 10% CCK‐8 reagent was premixed (MCE, China), and 100 µL of the mixed solution was added to each well and incubated for 1 h at 37 °C. The absorbance at 450 nm was measured with a plate reader (Bio‐Rad, USA).

### Enzyme‐Linked Immunosorbent Assay (ELISA)

The culture medium of EESCs from each group was collected and centrifuged at 500 × g for 5 min. HMGB1 concentrations in the culture media supernatant were measured by an ELISA kit (Thermo Fisher Scientific, USA) following the manufacturer's instructions.

### Chemotaxis Assay

Twenty‐four‐well transwell plates with 8 µm‐diameter filter membranes were utilized. Approximately 4 × 10^5^ M0 macrophages suspended in 200 µL of serum‐free medium were seeded into the upper chamber. The lower chamber was filled with 700 µL of DMEM supplemented with 15% FBS containing 3 × 10^5^ EESCs that had been treated with or without lactate or 2‐DG for 24 h. Following a 48 h of incubation period at 37 °C in a 5% CO_2_ atmosphere, the cells in the upper chamber were fixed in paraformaldehyde for 15 min and subsequently stained with 0.1% crystal violet for 15 min. The cells adhering to the filter membrane were examined and counted under an inverted microscope.

### Measurement of GSH Levels

The intracellular GSH levels were measured by a GSH assay kit obtained from Solarbio (Solarbio, China). The cells were lysed completely by subjecting them to four consecutive freeze‒thaw cycles. The cell lysate was subsequently mixed with the GSH reagent, and the absorbance of the resulting mixture was measured at 412 nm by a plate reader.

### Measurement of Intracellular Fe^2+^ Levels

FerroOrange (Dojindo, Japan) was used to measure intracellular Fe^2+^ levels following the manufacturer's instructions. Briefly, the cells were incubated with 1 µm FerroOrange in HBSS for 30 min in a cell incubator. Fluorescence images were obtained from three separate dishes per treatment by a Nikon confocal C2 fluorescence microscope (Nikon, Japan).

### MDA

The MDA content was assessed by an MDA assay kit (Beyotime, China). Treated cells were lysed with Western and immunoprecipitation (IP) lysis buffer, and the intracellular MDA content was determined according to the manufacturer's instructions.

### Liperfluo Staining

Lipid peroxidation was detected by Liperfluo (Dojindo, Japan). The cells were incubated with 1 µm Liperfluo for 30 min at 37 °C following the manufacturer's instructions. After the Liperfluo was removed, the EESCs were washed three times with Hank's balanced salt solution (HBSS; Gibco, China). Fluorescence microscopy (Nikon, Japan) was used to capture fluorescence images from three separate dishes for each treatment.

### Lactate Production Assay

The cells were cultured to ≈40% confluency, after which the medium was changed to fresh culture medium. After 24 h, the culture medium was collected, and measurement of lactate production was performed by kits from BioVision (USA) according to the manufacturer's instructions. Cell counting was performed using a cell counter. One milliliter of extraction reagent was added to 5×10^6^ cells. The cells were disrupted by ultrasonic waves in an ice bath, followed by centrifugation at 12 000 × g for 10 min at 4 °C. The supernatant was then collected for testing.

### RNA m6A Quantification

Total RNA was extracted with TRIzol, and RNA quality was assessed by a NanoDrop (Thermo Fisher Scientific, USA). Total RNA m6A modification levels were determined by the EpiQuik m6A RNA methylation quantification kit (p‐9005; Epigentek Group Inc., USA). Briefly, 200 ng of RNA and m6A standards were coated onto assay wells, and the plates were subsequently washed. M6A RNA capture antibody and detection antibody were added successively. Finally, developer was added and incubated for 10 min at room temperature in the dark. The m6A levels were quantified colorimetrically, and the absorbance of each well was read at a wavelength of 450 nm. Then, calculations were performed based on the standard curve.

### MeRIP‒qPCR

Intact total RNA was extracted with a centrifugation column (MiniBEST Universal RNA Extraction Kit; Takara, Japan), and the extracted mRNA was further purified by the polyATtract mRNA Isolation System (Promega Corp., USA). m6A RNA immunoprecipitation was subsequently performed with a Magna MeRIP m6A kit (17–10, 499, Millipore, USA) according to the manufacturer's instructions.

### ChIP‒qPCR

For DNA immunoprecipitation, a DNA ChIP Assay Kit (CST, USA) was utilized. The cells were fixed with 4% formaldehyde and quenched with glycine (0.2 mol L^−1^). After sonication, the chromatin was treated with protein A/G beads at 4 °C for 30 min. The samples were then incubated with anti‐H3K18la antibody (1:100; Cat. PTM‐1401RM, PTM BIO, China), anti‐HIF1A antibody, or normal rabbit IgG overnight at 4 °C. Finally, the DNA was released from the immunoprecipitates for RT‒qPCR.

### IF Analysis

Cells were fixed and blocked with 5% goat serum albumin (Beyotime Biotechnology, China) for 30 min. Tissue sections were incubated with specific primary antibodies (see Table 2) overnight at 4 °C, followed by incubation with fluorescently‐labeled secondary antibodies for 1 h at room temperature. Fluorescence images were acquired using a Nikon fluorescence microscope (Tokyo, Japan).

### RNA Stability Assay

The cells were treated with Actinomycin D (Biofount, China) at 5 µg mL^−1^. After incubation for 0, 3, 6, 12, or 24 h, the cells were collected and RNA was extracted for qRT‐PCR as described above. The mRNA degradation rate was estimated according to published protocols. The degradation rate of RNA (K) was estimated by equation. The RNA life time (*t*_1/2_) can be calculated from the degradation rate as follows: *t*_1/2_ = ln_2_K.

### Animal Experiments

Six‐ to eight‐week‐old C57BL/6 female mice were purchased from the Animal Center at Harbin Medical University and housed in the animal facility. A mouse model of endometriosis was established as previously described.^[^ ^28^ ^]^ In brief, 17β‐estradiol‐3‐benzoate (30 µg kg^−1^, Sigma, USA) was administered to each donor mouse daily for 3 days. The uterine horns from the donor mice were minced into 1 mm^3^ fragments and immediately injected into the peritoneal cavity of two recipient mice with an 18‐gauge needle. Endometriotic lesions were allowed to establish for 5 days before treatment with erastin or 2‐DG. The mice were randomly sorted into four groups (*n* = 6): vehicle + PBS group, erastin (20 mg kg^−1^ d^−1^) + PBS group, vehicle + 2‐DG (100 mg kg^−1^ d^−1^) group, and 2‐DG + erastin group. The mice were sacrificed on Day 15, and endometriotic lesions were collected. The length and width of the lesions were measured with a Vernier caliper, and the volume of the lesions was calculated by the prolate ellipsoid geometric model: (length × width^2^) × 0.52. Ethical approval for all animal research was granted by the Institutional Animal Research Ethics Committee of Harbin Medical University.

### Statistical Analysis

Each experiment was conducted independently, with a total of three replicates. The data are shown as the means ± standard deviations (SDs). The statistical analyses were performed with Prism 8 software. The data between two groups were analyzed by two‐tailed Student's *t*‐test, and one‐way ANOVA was used to compare the means of more than two groups. Differences were considered significant at *p* < 0.05 (^*^ *p* < 0.05, ^**^ *p* < 0.01, and ^***^ *p* < 0.001; *p* ≥ 0.05 was considered not significant (NS)).

### Ethical Statement

The studies using human tissue samples were approved by the Ethics Committee of the Second Affiliated Hospital of Harbin Medical University (Number: KY2020‐030). Animal experiments were approved by the Animal Center at Harbin Medical University (Number: YJSDW2022‐161) and were performed with the provisions of the Declaration of Helsinki of 1975.

## Conflict of Interest

The authors declare no conflict of interest.

## Author Contributions

Z.L., J.L., and Y.G. contributed equally to this work. Z.L., Y.G., and Z.Z. were responsible for the experimental design. Z.L. and J.L. performed the experiments. H.Z., H.W., Z.L., and Y.C. were involved in the acquisition of tissues. Z.L., J.L., and Y.G. were responsible for analyzing and interpreting the data. Z.L. and Z.Z. wrote and edited the manuscript. All authors have read and approved the final submitted manuscript.

## Supporting information



Supporting Information

## Data Availability

Data sharing is not applicable to this article as no new data were created or analyzed in this study.
