# Supplementary material for: Elevated Histone Lactylation Mediates Ferroptosis Resistance in Endometriosis Through the METTL3‐Regulated HIF1A/HMOX1 Signaling Pathway
Source: Adv Sci (Weinh). 2025 Jun 10;12(31):e08220. doi: 10.1002/advs.202408220 (PMC12376633; doi:10.1002/advs.202408220)
Supplement: Supplementary file 1 — Supporting Information [file ADVS-12-e08220-s001.docx]

**
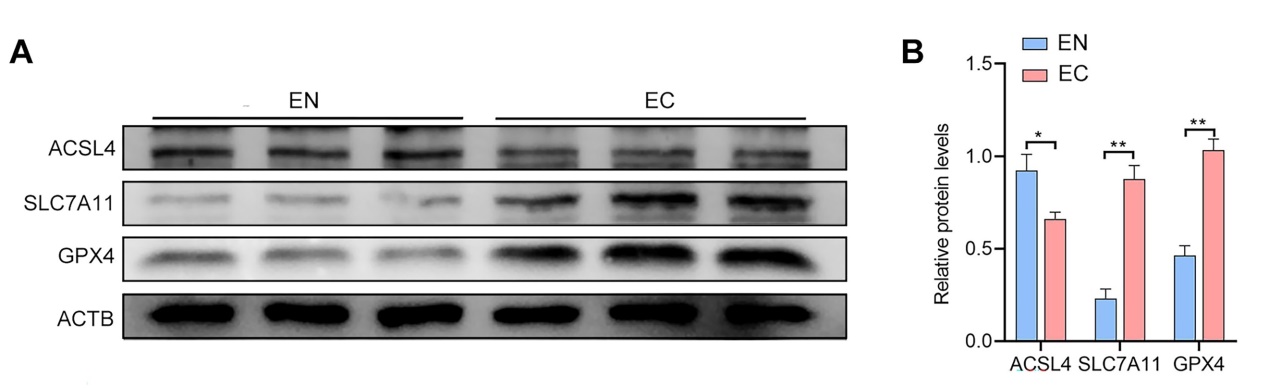
**

**Figure S1.** Ferroptosis resistance occurs in EMs. A, B)Western blot analysis was performed to detect the protein expression levels of ACSL4, SLC7A11, and GPX4 in EN and EC tissues.


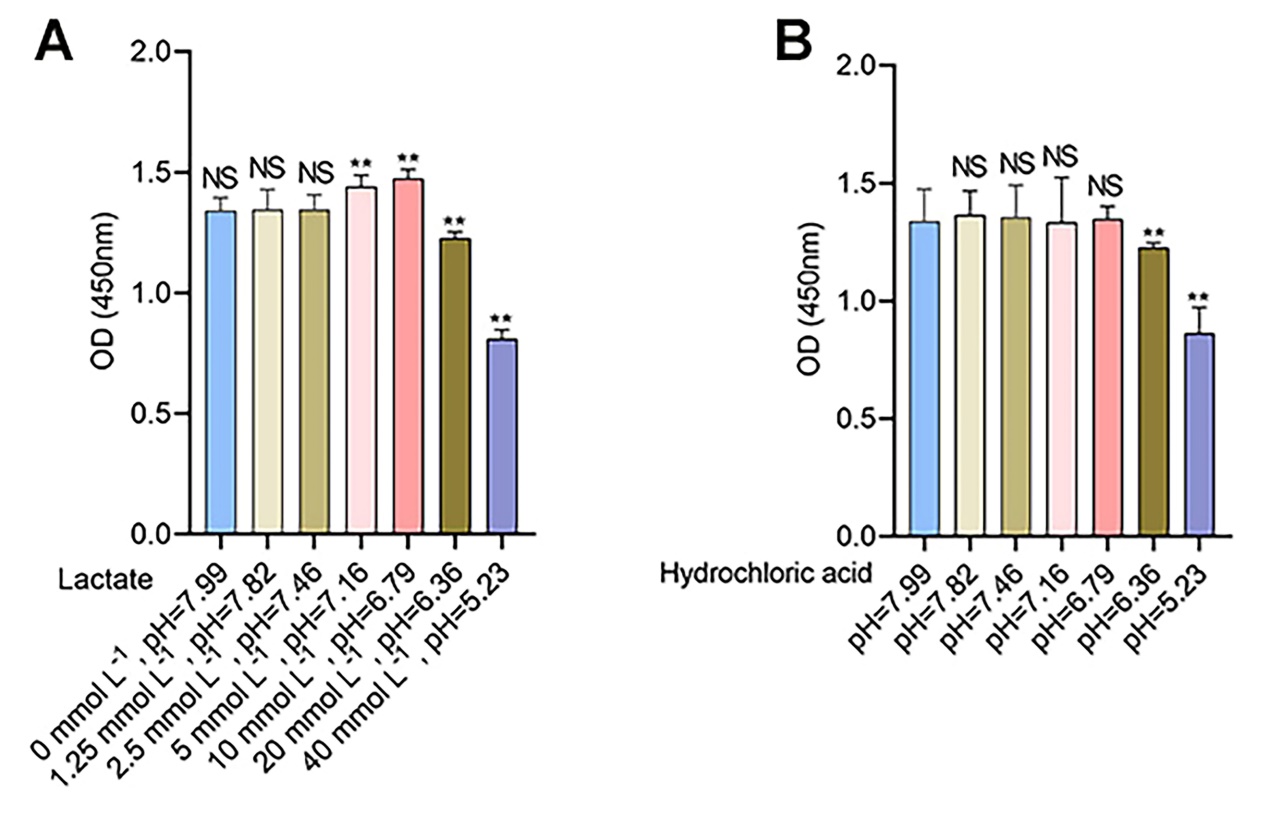


**Figure S2.** Effect of pH on the viability of EESCs. A)The impact of varying concentrations of lactate on cell viability. B) The effect of different pH values of hydrochloric acid on cell viability.


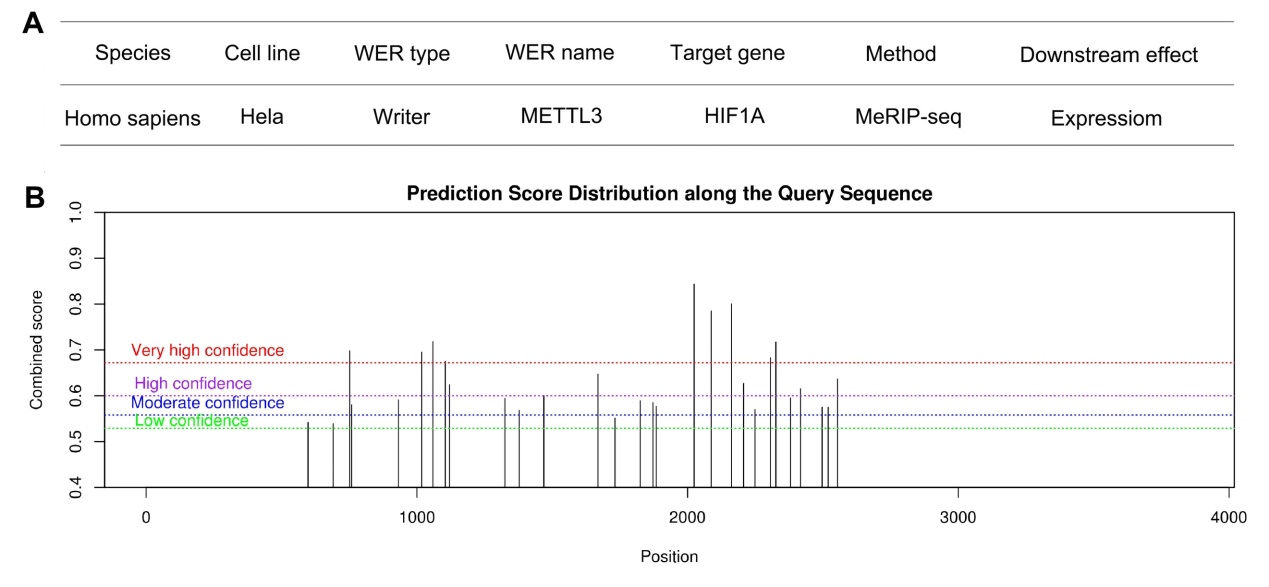


**Figure S3.** HIF1A may be a downstream target of METTL3. A) Candidate genes regulated by METTL3 were identified via the m6A2Target website. B) Potential m6A targets were investigated via the SRAMP website.


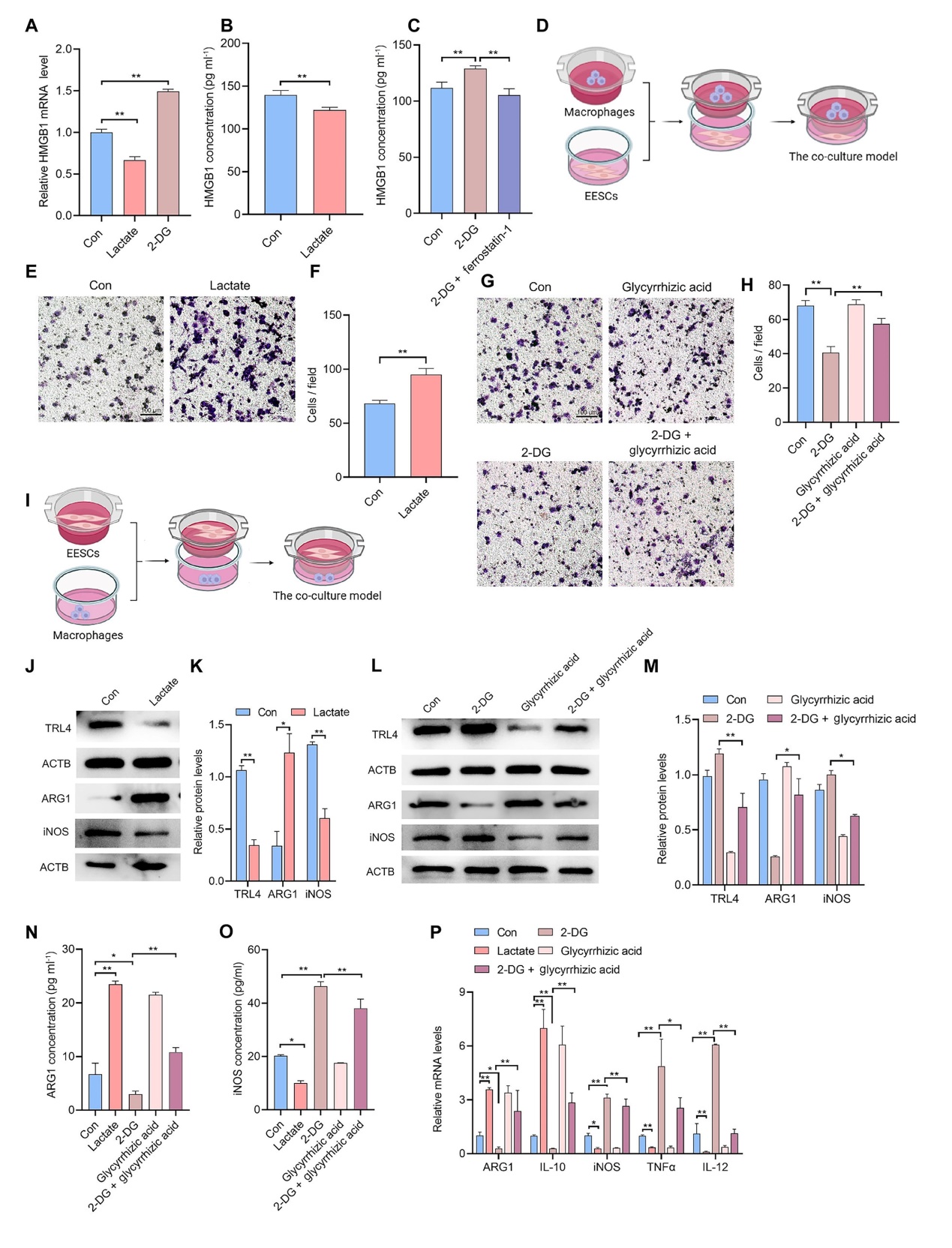


**Figure S4.** Ferroptosis resistance in EESCs modulates macrophage recruitment and polarization via HMGB1. A) HMGB1 expression in EESCs treated with 10 mmol L^-1^ lactate or 20 mM 2-DG for 24 h was tested by qRT-PCR. B) HMGB1 release from EESCs treated with 10 mmol L^-1^ lactate for 72 h was measured by ELISA. C) HMGB1 release from EESCs treated with 20 mM 2-DG or/and 5 μM ferrostatin-1 for 72 h was measured by ELISA. D) Schematic illustration of the co-culture setup used for panels E–H. M0 macrophages were seeded in the upper chamber, while EESCs were cultured in the lower chamber. E) EESCs were stimulated with or without lactate for 24h and subsequently co-cultured with macrophages for 48 h. The chemotactic ability of macrophages in the upper chamber was then assessed (scale bar = 100 μm). F) Macrophage counts per field. G) EESCs were stimulated with or without 2-DG or/and gycyrrhizic acid for 24h and subsequently co-cultured with macrophages for 48 h. The chemotactic ability of macrophages in the upper chamber was then assessed (scale bar = 100 μm). H) Macrophage counts per field. I) Schematic diagram of the co-culture system utilized for panels J–P, with EESCs placed in the upper chamber and M0 macrophages in the lower chamber. J-M) Western blot analysis was used to detect TLR4, ARG1, and iNOS protein levels in macrophages after co-cultured with EESCs for 48 h. J, K) The EESCs were pre-stimulated with or without lactate for 24h. L, M) The EESCs pre-stimulated for 24 h under the following conditions: Con, 2-DG, glycyrrhizic acid, 2DG + glycyrrhizic acid. N, O) ELISA assays was conducted to detect ARG1 (N) and iNOS (O) release levels from macrophages after co-cultured with EESCs for 72 h.The EESCs were pre-treated for 24 h under the following conditions: Con, lactate, 2-DG, glycyrrhizic acid, 2DG + glycyrrhizic acid. P) qRT-PCR was conducted to detect ARG1, IL-10, iNOS, TNFα, and IL-12 expression in macrophages after co-cultured with EESCs for 24 h.The EESCs were pre-treated for 24 h under the following conditions: Con, lactate, 2-DG, glycyrrhizic acid, 2DG + glycyrrhizic acid. Error bars represent the mean ± SD. Two-tailed Student’s *t* test was used to compare the means of two groups; One-way ANOVA was used to compare the means of more than two groups; ^*^*P* < 0.05; ^**^*P* < 0.01.


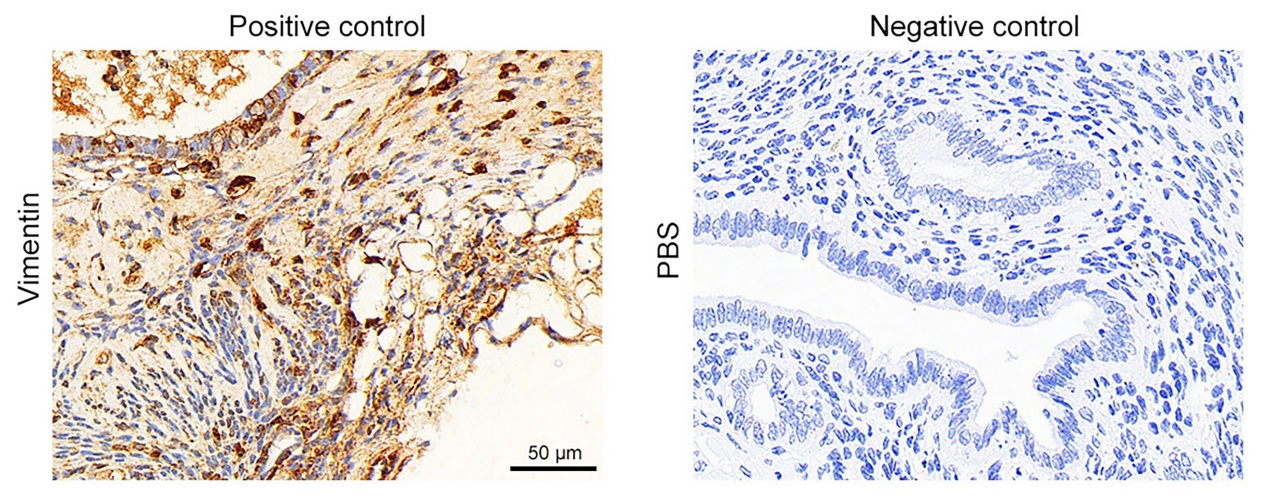


**Figure S5.** Immunohistochemical positive and negative controls. A) Vimentin immunohistochemistry served as the positive control. B) PBS was used for immunohistochemistry and served as the negative control.
